# Zinc β-Diketonates with Donor-Acceptor Ligands: Synthesis and Comprehensive Structural, Thermal, and Photophysical Characterization

**DOI:** 10.3390/molecules30224325

**Published:** 2025-11-07

**Authors:** Ahmad Daher, Manjiri Choudhari, Thomas Roland, Vincent De Waele, Stéphane Daniele

**Affiliations:** 1 Catalyse, Polymérisation, Procédés et Matériaux (CP2M), Chimie École Supérieure de Chimie Physique Électronique de Lyon (CPE Lyon), CNRS UMR 5128, Université de Lyon, F-69100 Villeurbanne, France; ahmad.daher@univ-lyon1.fr; 2 Univ. Lille, CNRS, UMR 8516, LASIRE-Laboratoire de Spectroscopie pour les Interactions, la Réactivité et l’Environnement, 59000 Lille, France; choudhari.manjiri@univ-lille.fr (M.C.); thomas.roland@univ-lille.fr (T.R.); vincent.de-waele@univ-lille.fr (V.D.W.)

**Keywords:** zinc complexes, β-diketonates, heteroleptic complexes, ultrafast spectroscopy, excited-state dynamics, structure-property relationships

## Abstract

We report the synthesis, spectroscopic, structural, and ultrafast photophysical investigation of a series of homoleptic and heteroleptic Zn(II) complexes based on the donor-acceptor β-diketonate ligand 4,4,4-trifluoro-1-phenylbutane-1,3-dione. Mass spectrometry, infrared, and NMR analyses confirm complexation and indicate possible fragmentation pathways involving the sequential loss of β-diketonate ligands. Single-crystal X-ray diffraction revealed that all complexes adopt monomeric octahedral geometries, with the ancillary nitrogen-based ligands introducing variable distortions. Thermal analyses confirmed that the complexes are non-volatile and have an onset >250 °C, with thermal decomposition primarily to ZnO and ZnF_2_. Complexes with aromatic Lewis base led to higher residue percentages, likely due to the final graphitic carbon content. UV-Vis absorption and femtosecond transient absorption spectroscopy demonstrate that the chelated β-diketonate ring serves as the main optically active chromophore, a property unaffected by the nitrogen ligands. The free ligand undergoes rapid internal conversion, whereas coordination to Zn stabilizes the triplet state via LMCT, producing long-lived and chemically reactive species relevant to dissociation processes. This study demonstrates how tailored ligand environments can be exploited to tune excited-state properties, offering a rational framework for the design of functional precursors suitable for nonlinear photolysis and advanced nanomaterial synthesis.

## 1. Introduction

β-Diketonate ligands (R^1^COCHR^3^COR^2^) are among the most versatile and widely exploited classes of precursors for metal-organic chemical vapor deposition (MOCVD) of thin films [1,2,3]. Their tunable volatility, coordination flexibility, and favorable decomposition behavior make them especially suited for gas-phase delivery of metal centers [4]. For example, fluorinated β-diketonate complexes M(tfac)_2_(TMEDA) (M = Fe, Ni, Cu, and Zn) have recently been demonstrated as effective MOCVD precursors for the deposition of both metal and metal oxide films, with controlled film quality (e.g., well-oriented ZnO thin films) and composition, thereby highlighting the critical role of ligand design in optimizing volatility and film quality [5]. Previous work from our group has shown that γ-substitution (R^3^ nature) can be exploited to tune the stability and degradation pathways of titanium β-diketonate precursors, paving the way for the rational design of improved molecules for TiO_2_ thin-film deposition [6].

ZnO is an attractive target material due to its wide band gap, biocompatibility, piezoelectric properties, relevance in microelectromechanical systems (MEMSs) and sensors, and in photonics. β-Diketonate ligands are particularly suitable for Zn(II) chemistry because they act as chelating donors that promote electron delocalization when coordinated to metal ions. This occurs through the formation of Zn^2+^-ligand donor-acceptor complexes that promote low-energy ligand-to-metal charge transfer (LMCT) processes [7], enhancing multiphoton absorption and enabling precise laser-induced material growth.

While MOCVD remains the dominant approach for ZnO film growth [8,9], the development of next-generation micro- and nanotechnologies increasingly demands 3D, high-resolution, and environmentally benign fabrication techniques. In addition, minimizing the use of environmentally costly elements is also considered a necessity [10,11,12,13,14]. Conventional microsynthetic strategies, however, are limited to 2.5D processing and cannot achieve true 3D structuring at the sub-micrometer scale [15,16]. High-throughput additive manufacturing is often restricted either to feature sizes of tens of micrometers or to organic polymers, limiting its applicability for functional inorganic materials [17,18,19]. To overcome these limitations, ultrafast laser-induced deposition (ULD) has emerged as a promising alternative which requires the design of molecular precursors that efficiently absorb multiphotonic excitation and undergo controlled, laser-triggered decomposition. Nevertheless, it remains limited to a narrow range of ligands [20,21].

Laser excitation induces complex coherent superpositions of excited states that evolve on femtosecond timescales through internal conversion or direct relaxation to the ground state, often accompanied by excess vibrational energy. These processes depend on the metal center, ligand type, and excitation conditions. They can be tuned by adjusting the coordination environment to control solubility, electronic and optical properties, and decomposition pathways. ULD offers a promising alternative, enabling the localized and additive growth of inorganic materials under mild conditions providing a flexible route for 3D fabrication [22,23,24,25]. The main challenge for this approach is the design of molecular precursors that efficiently absorb multiphotonic excitation and undergo controlled degradation under nonlinear, laser-driven conditions. A fundamental understanding of their ultrafast photophysics is therefore essential [9,10,11,12,13].

To date, such β-diketonate architectures have not been systematically investigated using femtosecond spectroscopic techniques. In this work, we report the synthesis and full characterization of a series of homoleptic and heteroleptic β-diketonate Zn(II) complexes bearing a donor-acceptor β-diketonate ligand. Their structural, thermal, and electronic properties were examined by mass spectrometry, NMR, single-crystal X-ray diffraction, thermal analysis, and UV-Vis spectroscopy. Femtosecond spectroscopy was further employed to probe their excited-state dynamics, providing insights into their photophysical behavior and decomposition mechanisms. We further investigate the influence of different ancillary ligands on the stability, optical properties, and excited-state relaxation pathways. This comprehensive study establishes important structure-property relationships for Zn(II) β-diketonates, laying the groundwork for their future application as ZnO precursors and functional materials.

## 2. Results and Discussion

### 2.1. Synthesis

**Scheme 1:**
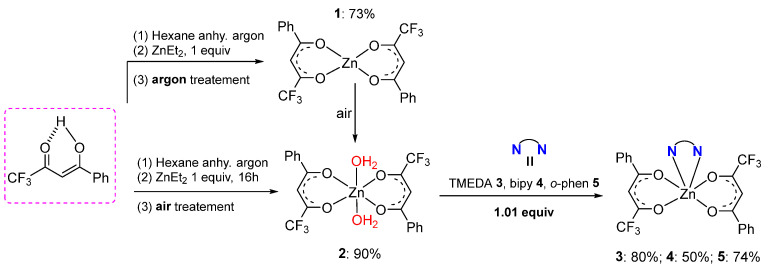
Synthesis of the zinc complexes **1**–**5** starting from ligand **L^1^H**.

The zinc β-diketonate complexes were synthesized via a Brønsted acid-base reaction between the dissolved, enolic form of the ligand **L^1^H** and ZnEt_2_ in a 2:1 molar ratio under argon. Ethane was the only byproduct in the reaction to give **1** in 73% [26] (Scheme 1). Exposing the reaction mixture to air yielded the hydrated product **2** (90%), while the addition of nitrogen-based Lewis bases (TMEDA, bipy, and *o*-phen) led to the formation of complexes **3**–**5** in moderate to excellent yields (50–90%). Water replacement to these heteroleptic complexes was driven by entropy. Complex **4** (50%) was obtained in slightly lower yields in comparison to its analogs due to the partial loss during the purification process when removing the excess bipyridine.

### 2.2. Mass Spectrometry: Electro-Spray Ionization (ESI)

**Scheme 2:**
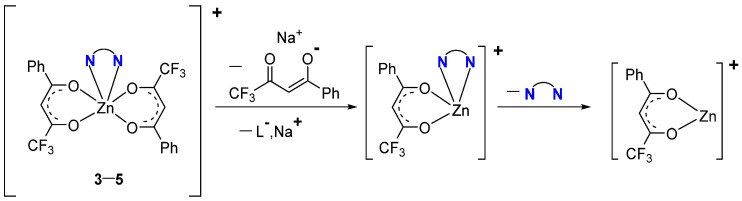
Proposed fragmentation of the heteroleptic complexes **3**–**5** (1 mg/mL).

ESI mass spectra of all complexes in absolute EtOH (Figures S9–S12, Supplementary Materials) showed the parent [M + Na]^+^ ions along with characteristic fragmentation patterns. A clear peak corresponding to the loss of one β-diketonate ligand was observed, followed by the loss of the ancillary Lewis base in heteroleptic complexes **3**–**5**. The peaks for the free, protonated Lewis bases were also detected, supporting the proposed coordination. These fragmentation pathways are summarized in Scheme 2.

### 2.3. NMR and ATR-FTIR Spectroscopy

All complexes exhibit solubility in polar organic solvents. The characterization by ^1^H and ^19^F NMR spectroscopies (see spectroscopic characterizations in Supplementary Materials for spectra) confirms the coordination for each of the β-diketonate ligands and the Lewis base with zinc for the heteroleptic complexes. Interestingly, coordination induces only minor chemical shift changes in the ^1^H NMR spectra.

The ATR-FTIR data show that **L^1^H** exhibits a C=O stretching band at 1600 cm^−1^, while neither an O-H band at around 3300 cm^−1^ nor a C=C band at around 1550 cm^−1^ are present. This implies that the undissolved ligand is predominantly in its ketone form [27]. ATR-FTIR spectra further support the formation of the complexes, as the main Zn-O bond stretching vibration bands appear in the usual range. For example, in complex **2**, the ligand undergoes enolization prior to coordination. The coordination of the Zn center to the enolate form of the diketone gives rise to characteristic Zn-O enolate bands at 582 cm^−1^ and 638 cm^−1^ [28,29] (see Figures S1–S5 in the Supplementary Materials for individual spectra). The C-O stretching band appears at 1311 cm^−1^ and the broad band at 3363 cm^−1^ corresponds to the O-H vibration of H_2_O. In complexes **3**–**5**, the O-H band disappears as H_2_O is replaced by the Lewis bases. C=C stretching bands remain relatively unchanged across all complexes (1573–1577 cm^−1^), while the C-O band in **5** shifts to 1286 cm^−1^, likely due to electronic transfer from Zn(II) (d^10^) to oxygen atoms from the inductive and/or electrostatic effects associated with coordination.

### 2.4. Single-Crystal X-Ray Diffraction

**Figure 1:**
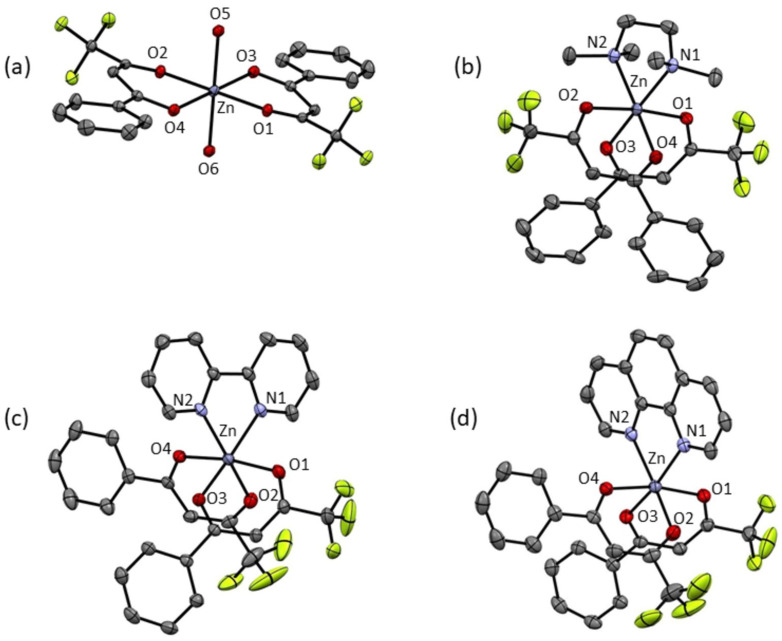
ORTEP crystal structure of (**a**) [Zn(L^1^)_2_(H_2_O)_2_] (**2**); (**b**) [Zn(L^1^)_2_(TMEDA)] (**3**); (**c**) [Zn(L^1^)_2_(bipy)] (**4**); and (**d**) [Zn(L^1^)_2_(*o*-phen)] (**5**). (Ellipsoids with 50% probability and for the sake of clarity, carbon and fluorine atoms are in gray and yellow, respectively.

Single crystals of complexes **2**–**5** were formed via the slow evaporation of saturated CH_3_CN solutions at room temperature (Figure 1a–d). Selected bond lengths and angles are summarized in Table 1. All complexes are monomeric, with zinc atoms in a hexa-coordinated octahedral geometry (Figure 1a–d) [30] with six oxygen atoms O1–O6 in complex **2** (including two H_2_O molecules), or, as in complexes **3**–**5**, with four oxygen atoms from diketones and two nitrogen atoms N1 and N2 provided by the ancillary ligand. In **2**, each donating atom is positioned opposite to its equivalent at 180° (e.g., O1-Zn-O2), with the two β-diketonate ligands in a trans configuration. In contrast, heteroleptic complexes **3**–**5** adopt cis configurations due to the chelating nature of the LB, resulting in distorted octahedral geometries [5,31]. The extent of the distortion correlates with ligand rigidity: TMEDA in **3** produces minimal distortion (O1-Zn-O2 = 175.53°), while bipy and *o*-phen in **4** and **5** induce larger deviations from ideal bond angles (O1-Zn-O2 = 86° and 84°).

The M-O bonds involving the same donor atom (e.g., Zn-O1 and Zn-O2 adjacent to the carbon-bearing CF_3_ group) are nearly identical in length, except in complex **4**, where the bond is about 0.04–0.05 Å longer. In general, M-O bonds near the C-CF_3_ moiety are shorter than those closer to C-Ph (Zn-O3 and Zn-O4). This difference might be due to the electron-withdrawing effect of the CF_3_ moiety. In complex **2**, the coordinated water molecules engage in multiple O···H and O···O contacts, while additional C-H···F interactions occur between the phenyl hydrogens and the CF_3_ groups of the β-diketonate ligands. Complex **3** displays C-H···F contacts involving both the methyl groups of the nitrogen of TMEDA and fluorine of the β-diketonate ligands. In complex **4**, pyridyl and phenyl C-H groups interact weakly with fluorine and oxygen atoms (C-H···F and C-H···O), along with other non-specific C···F contacts, forming a compact packing arrangement. Finally, complex **5** exhibits C-H···F, C-H···O, and O···C van der Waals contacts between the *o*-phenanthroline hydrogens and β-diketonate fluorine moieties, which together reinforce the solid-state structure. The obtained data for bond lengths, angles, and bond interactions are remarkably close to those reported for the crystal structures of fluorinated β-diketones coordinated with TMEDA, such as [Zn(tfac)_2_(TMEDA)] [5] and [Zn(hfac)_2_(TMEDA)] [32].

### 2.5. Thermal Analysis

**Figure 2:**
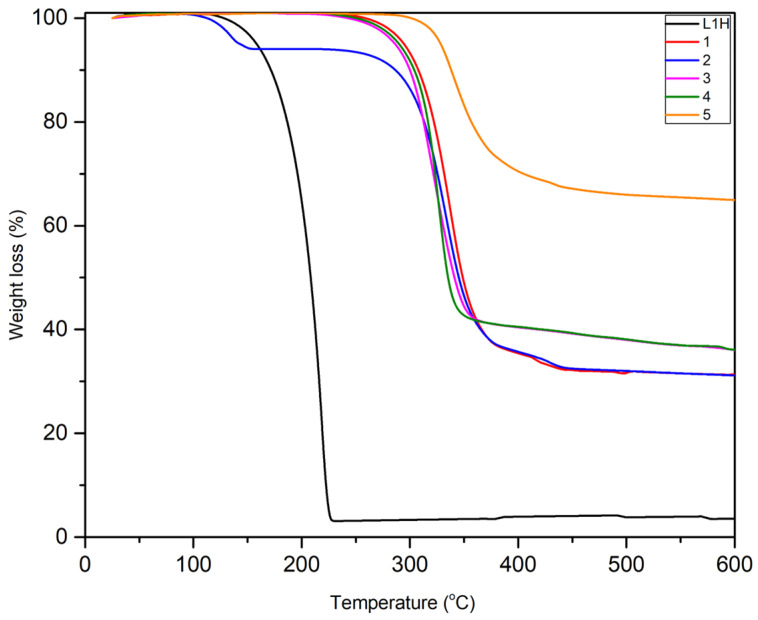
TGA curves of **L^1^H** and complexes [Zn(L^1^)_2_]_m_ (**1**), [Zn(L^1^)_2_(H_2_O)_2_] (**2**), [Zn(L^1^)_2_(TMEDA)] (**3**), [Zn(L^1^)_2_(bipy)] (**4**), and [Zn(L^1^)_2_(*o*-phen)] (**5**).

Thermogravimetric analysis (TGA) was performed from room temperature to 600 °C under a nitrogen atmosphere to investigate thermal stability and volatility (Figure 2). **L^1^H** displays volatility with an onset of 120 °C, related to its relatively low molecular weight, despite potential π-stacking interactions. Coordination with zinc enhances thermal stability, with all complexes showing weight loss above 250 °C. Complex **2** exhibits an initial 6% mass loss at 120 °C, corresponding to H_2_O release [29]. The anhydrous complexes **3** and **4** show similar volatility to **2** but undergo single-step decomposition. Unusually, the Lewis base nature has limited effect on thermal stability. Only when *o*-phen was used in complex **5** was an increase in the decomposition temperature by ~50 °C observed, concurrently yielding a higher residual mass (66%) compared to bipy (46%) and TMEDA (38%). For complexes **1**–**3**, the final residues at 600 °C can be attributed mainly to ZnO and ZnF_2_, as the experimental values are close to the sum of their theoretical residues in addition to small amounts of char, which is common in such TGA analyses [33]. ZnF_2_ is known to form from zinc fluorinated β-diketonate complexes (e.g., hfacacH) [4]. In contrast, for complexes **4** and **5**, the addition of aromatic ligands such as bipy and *o*-phen leads to higher experimental residues due to the higher carbon content (Table 2).

### 2.6. Femtosecond Transient Absorption Spectroscopy (TAS)

**Figure 3:**
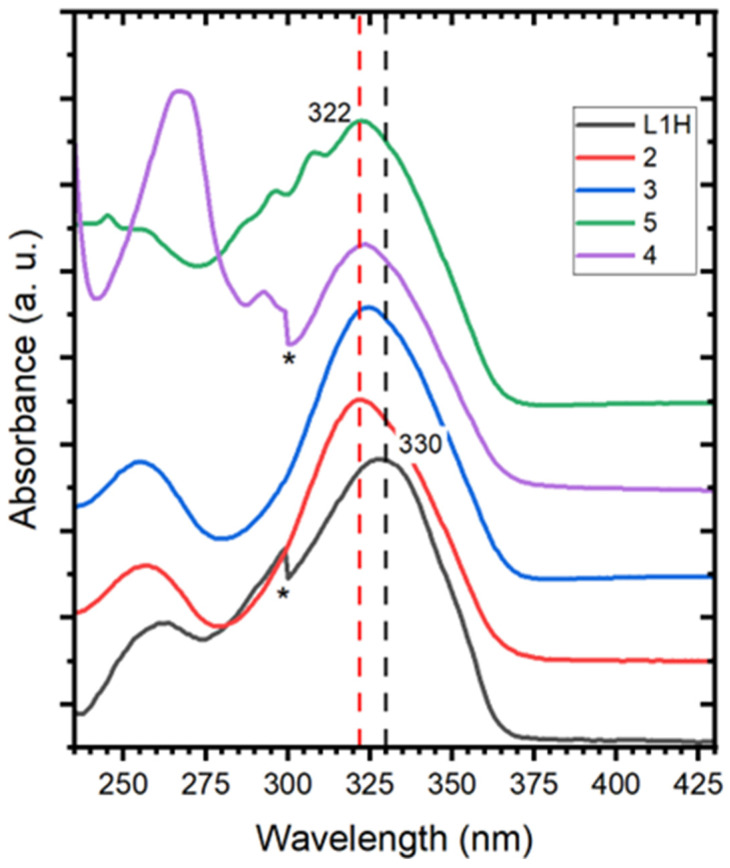
UV-vis absorption spectra of compounds **L^1^H** and complexes [Zn(L^1^)_2_(H_2_O)_2_] (**2**), [Zn(L^1^)_2_(TMEDA)] (**3**), [Zn(L^1^)_2_(bipy)] (**4**), and [Zn(L^1^)_2_(*o*-phen)] (**5**) in absolute EtOH (10^−5^ M). The spectra have been vertically shifted for clarity. The stars mark an artifact from the spectrometer. Dashed lines indicate the values of maximum absorbance.

The ground-state electronic transitions of **L^1^H** and of **2**–**5** were studied using steady-state absorption spectroscopy (Figure 3). **L^1^H** exhibits a strong absorption band at 330 nm and a secondary peak at 260 nm, consistent with related β-diketones such as dibenzoylmethane (DBM) [34]. The intense band at 330 nm is assigned to a π→π* (S_0_-Sₙ) transition associated with the chelated ring of **L^1^H**. In Figure 3, the absorption band of **2**–**5** around 330 nm is retained but exhibits a slight blue shift of approximately 8 nm. Additionally, in **4** and **5**, absorption bands corresponding to the bipy (265 nm) and *o*-phen (280–320 nm) moieties are observed. The UV-Vis spectra of the complexes are dominated by the absorption of **L^1^H**, which has a π→π* character, while contributions from the ancillary ligands are observed only below 300 nm.

**Figure 4:**
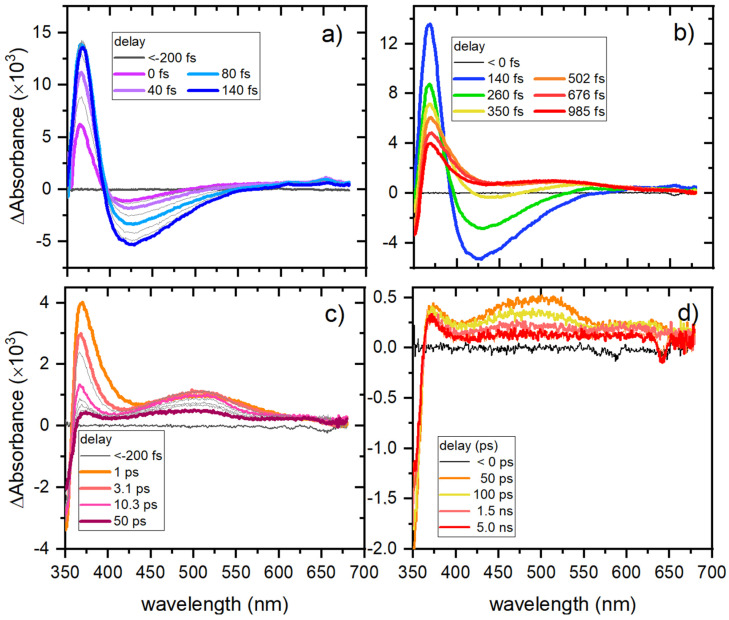
Transient absorption spectra of compounds **L^1^H** recorded in absolute EtOH with a pump excitation λ_pump_ = 320 nm and selected pump-probe delays in the ranges of (**a**) 0 fs to 140 fs, (**b**) 140 fs to 965 fs, (**c**) 1 ps to 50 ps, and (**d**) 50 ps to 5.0 ns.

UV-Vis transient absorption experiments were performed to characterize the excited states formed upon photoexcitation of the compounds, focusing on the influence of the ancillary ligands. All transient spectra were recorded in EtOH following excitation at 320 nm, in resonance with the optically active π→π* transition. The results obtained for **L^1^H** are shown in Figure 4. Between 0 and 140 fs, an ESA band develops at 375 nm alongside a stimulated emission (SE) feature at 425 nm, indicating the population of the π→π* excited state. Within less than 1 ps, this state converts to a new transient species characterized by a reduced ESA intensity below 400 nm and the appearance of a broad ESA band at 525 nm, while the SE disappears. A negative feature below 370 nm corresponds to ground-state bleach (GSB). Subsequent evolution between 1 and 10 ps involves decay of the ESA below 400 nm and partial GSB recovery, consistent with the vibrational cooling of a hot ground state. Complete recovery of the ground state occurs after several hundred picoseconds.

These findings confirm that excitation at around 320 nm populates the π→π* state localized on the chelated ring of **L^1^H**, which acts as the principal chromophore for all complexes. The observed dynamics for **L^1^H** are consistent with previous studies on β-diketones, following the sequence S_2_ (π→π*) → S_1_ (n→π*) → S_0_, dominated by ultrafast internal conversion to the ground state with minor intersystem crossing contributions.

Although the initial excitation pathway of **L^1^H** (S_0_ → S_2_ → S_1_) mirrors that of other β-diketones, its subsequent deactivation is profoundly different. In systems like DBM and benzoylacetone, the S_1_ state can split into two pathways, where one leads to a significant yield of a non-chelated enol (NCE) photoproduct via rotamerization [35,36]. In stark contrast, our transient absorption data for **L^1^H** shows no evidence of this NCE species. The dynamics are instead characterized by the complete recovery of the ground-state bleach, indicating that deactivation is dominated overwhelmingly by direct internal conversion back to the original chelated enol ground state. This demonstrates that the specific donor-acceptor structure of **L^1^H**, featuring the strong electron-withdrawing -CF_3_ group, effectively suppresses the rotamerization channel, resulting in a ‘purified’ deactivation mechanism.

**Figure 5:**
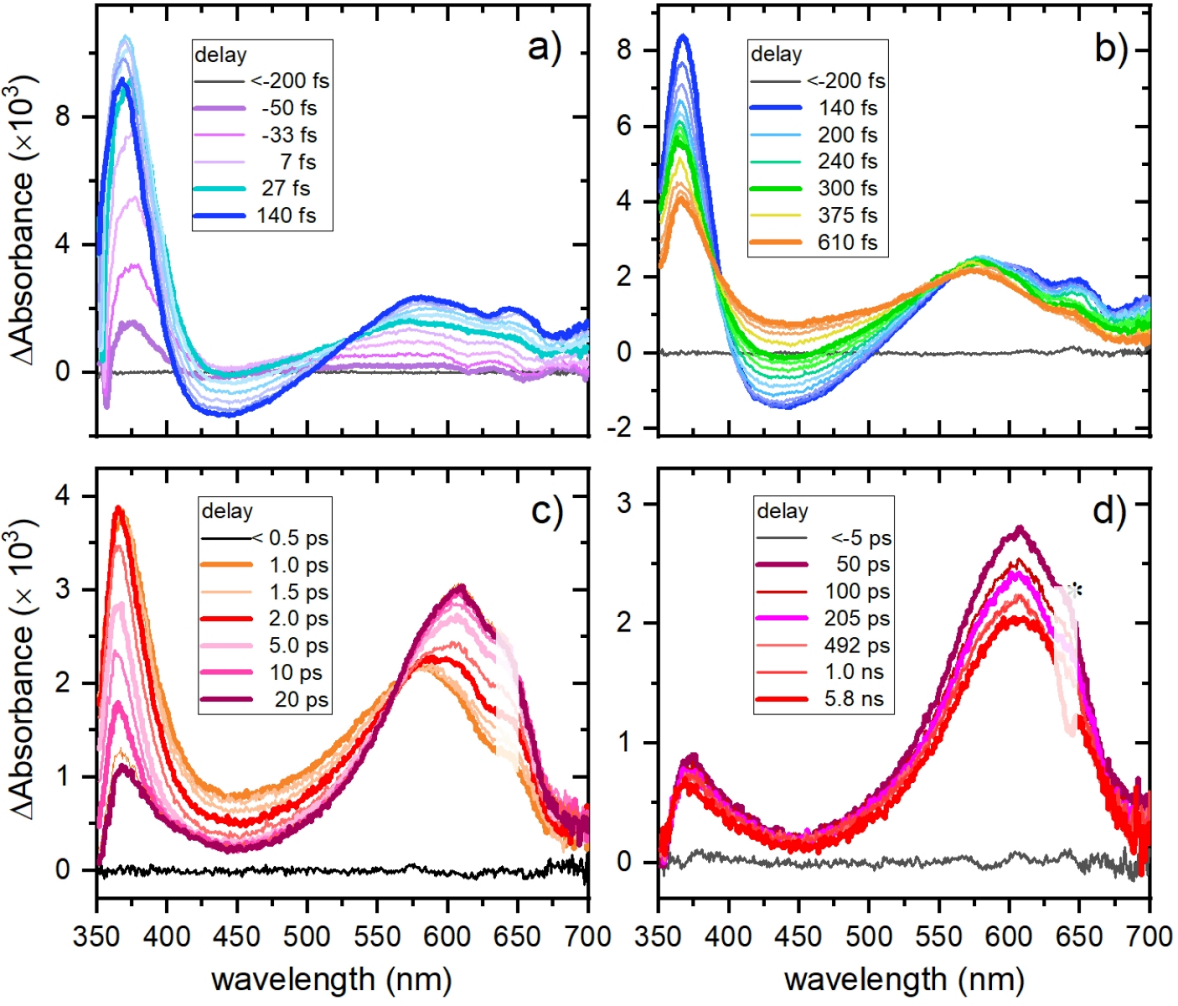
Transient absorption spectra of compound [Zn(L^1^)_2_(H_2_O)_2_] (**2**) recorded in absolute EtOH with a pump excitation λ_pump_ = 320 nm and selected pump-probe delays in the ranges of (**a**) −50 fs to 140 fs, (**b**) 140 fs to 610 fs, (**c**) 0.5 ps to 20 ps, and (**d**) 50 ps to 5.0 ns. (The white area masks the residual light from the second order dispersion of the pump pulses).

The transient absorption spectra recorded for **2**–**5** under identical conditions exhibit a similar qualitative behavior (Figure 5 and Figures S14–S16 in the Supplementary Materials) but differ from that of **L^1^H**. In Figure 5a, the π→π* excited state is identified by its ESA at 370 nm and SE at around 425 nm. Compared to **L^1^H**, however, a strong new ESA contribution appears at 550–600 nm. Within less than 1 ps, the ESA and SE both decrease, an isosbestic point emerges at 400 nm, and the red tail of the ESA slightly decays at 575 nm, reflecting an internal conversion from the π→π* state to a less-emissive excited state. The latter transforms into a third transient species (Figure 5c) with a distinct ESA maximum at 625 nm. This final species is long-lived, showing no spectral evolution on the nanosecond timescale, consistent with the formation of a triplet state. The decay times and the decay-associated spectra (see Figures S17–S20 in Supplementary Materials) show similar transient spectra and kinetic behavior for **2**–**5**, consisting in the following sequence:

$$S_{2}\overset{150-180\;fs}{\to }\;S_{1}\overset{5-6\;ps}{\to }\;T_{1}^{hot}\overset{50\;ps,\;500\;ps^{-1}\;ns}{\to }T_{1}\;$$

The photodynamic is mainly localized on one **L^1^** ligand. It starts with the ultrafast S_2_ to S_1_ internal conversion (t_1_ = 150–180 fs), followed by the S_1_-T_1_ internal conversion (t_2_ = 5–6 ps). Then the amplitude of the T_1_ absorption decays partially with a t_3_ = 50 ps and 500 ps^−1^ ns time constant. We assigned the slow dynamics (t_3_ and t_4_) to the vibrational relaxation and the conformational equilibration. The ancillary ligands exert minimal influence on the excited-state dynamics. We detect indeed only a small shift in the T_1_ absorption band (Figure S21) and in the kinetics. However, by comparing the amplitude of the S_2_ absorption with that of T_1_ in Figures S17–S20, we can notice that the relative final amount of triplet states varies with the ancillary ligands. The highest conversion is obtained for TMEDA (**3**), the lowest for *o*-phen (**5**).

The photophysical profile of **L^1^H** is fundamentally reconfigured upon coordination to Zn(II). The enhanced structural rigidity of the resulting complexes suppresses the ultrafast internal conversion that dominates the dynamics of the free **L^1^H**. This suppression enables a well-defined relaxation cascade through S_2_ and S_1_ states, culminating in efficient population of a long-lived triplet state (T_1_). This T_1_ state, which persists on the nanosecond to microsecond timescale and exhibits a red-shifted excited-state absorption, serves as the primary energy reservoir. This represents a fundamental functional shift: from a ligand that dissipates energy as heat, to a complex that stores energy in a persistent, chemically reactive triplet reservoir.

## 3. Materials and Methods

The FT-infrared spectra (FT-IR) of the ligand and complexes were recorded using a Nicolet iS50 FT-IR spectrometer (Thermo Fisher Scientific, Waltham, MA, USA). UV-visible absorbance measurements were performed using a Thermo Scientific Evolution 220 dual-beam spectrophotometer at a scan rate of 1 nm/s (Thermo Fisher Scientific, Waltham, MA, USA). ^1^H and ^19^F NMR spectra were acquired on a Bruker SampleXpress 300 MHz spectrometer (Bruker BioSpin, Ettlingen, Germany) (s = singlet, d = doublet, t = triplet, and m = multiplet). ESI mass spectra were recorded using an Impact II mass spectrometer (Bruker Daltonics, Billerica, MA, USA). Thermal behavior was analyzed using a METTLER TOLEDO TGA 2 Star System (METTLER TOLEDO, Columbus, OH, USA) under a nitrogen atmosphere at a scan rate of 5 K min^−1^. Femtosecond transient absorption measurements have been conducted using the setup described in [37]. Briefly, the system consists of a 5 W Astrella laser delivering 35 fs pulses used to seed an OPA and a Helios UV-vis transient absorption spectrometer (Ultrafast Systems, Sarasota, FL, USA). All the measurements were performed in solution using a 1 mm flowing cell.

Single-crystal X-ray diffraction data of complexes **2**–**5** were measured using ω scans with Mo Kα radiation at 100 K. The diffraction pattern was indexed, and the total number of runs and images was based on the strategy calculation from the program CrysAlisPro system [38] (CCD 43.125a 64-bit, released 4 June 2024). The maximum resolution that was achieved was θ = 30.615° (0.70 Å). The unit cell was refined using CrysAlisPro (Rigaku Oxford Diffraction, Abingdon, UK, 2024) on 20,645 reflections (64% of the observed reflections). Data reduction, scaling, and absorption corrections were performed using CrysAlisPro. A multi-scan absorption correction (SCALE3 ABSPACK) was applied. The dataset is 100% complete to 30.615° in θ. The absorption coefficient (μ) is 0.945 mm^−1^ at λ = 0.71073 Å. Minimum and maximum transmissions are 0.768 and 1.000. Data were collected at 100 K. Cell parameters and orientation matrices were determined from 36 frames of 2D diffraction images. The data were corrected for Lorentz and polarization effects. The structure was solved using SHELXT (Sheldrick, Univ. of Göttingen, Germany) [39] and refined using SHELXL (Sheldrick, Germany) [40] within the Olex2 interface (Durham University, UK) [41].

ZnEt_2_ (96%, CAS no: 557-20-0) in PhMe solution (2 M) and **L^1^H** (99%, CAS no: 720-94-5) were purchased from the Thermo Fisher Scientific and Merck-Sigma Aldrich (Saint Louis and Burlington, MA, USA) companies, respectively, and used without further purification. Nitrogen-based LB: N,N,N′,N′-Tetramethylethylenediamine (TMEDA, 95%, CAS no: 612-00-3), bipy (99%, CAS no: 366-18-7), and *o*-phen.H_2_O (99%, CAS no: 66-71-7) were purchased from the Merck-Sigma Aldrich and Alfa Aesar (Haverhill, MA, USA) companies, respectively, and used without any further purification. Solvents like PhMe (99.8%, CAS no: 108-88-3), anhydrous n-hexane (95%, CAS no: 110-54-3), THF (99.8, CAS no: 109-99-9), and CAN (99.9%, 75-05-8) were bought from Merck in Lyon. Absolute ethanol (99.8%, CAS no: 64-17-5) was purchased from VWR, Fontenay-sous-Bois, France. Deuterated solvents were purchased from Merck: acetone-D6 (CAS 666-52-4, 99.9% D), chloroform-D1 (CAS 865-49-6, 99.8% D), and dichloromethane-D2 (CAS 1665-00-5, 99.8% D) for our experiments. Solvent removal was carried out using a rotary evaporator (Heidolph Instruments GmbH & Co. KG, Schwabach, Germany).

**L^1^H**: C_10_H_7_O_2_F_3_. 216 g·mol^−1^. ^1^H NMR (MHz, CD_2_Cl_2_, δ ppm): 7.97 (2H, d, *J* = 7.23 Hz, HC_ar_); 7.66 (1H, t, *J* = 7.38 H, HC_ar_); 7.53 (2H, t, *J* = 7.7 Hz, HC_ar_); 6.62 (1H, s, -CH-). ^19^F NMR (CD_2_Cl_2_, δ ppm): 76.86. FT-IR (cm^−1^): 3126 cm^−1^ (νCsp^2^/Csp^3^ - H), 1600 cm^−1^ (νC=O), 1480 cm^−1^. ESI (*m*/*z*): [M + H]^+^ = 217.04. UV-vis (Absolute EtOH): λ_1_max = 327 nm. Band gap = 3.79 eV.

**General procedure for the synthesis of the complexes 1–5**

The synthesis (via Brønsted acid-base reaction), characterizations and purification of the non-hydrated complex **1** was performed under argon, starting from ligand **L^1^H** (Scheme 1, left). The same reaction but treated under air led to the hydrated counterpart **2** (Scheme 1, right). Heteroleptic zinc complexes **3**–**5** were produced when nitrogen-chelating LB ligands such as TMEDA, bipy, or *o*-phen were added to the medium in a Zn/LB respective molar ratio of 1:1.

**[Zn(L^1^)_2_]_m_ (1)**

Under argon, 1 g of **L^1^H** (4.62 mmol) was vigorously stirred and dissolved in anhydrous n-hexane (20 mL). A 3 mL PhMe solution of ZnEt_2_ (2.31 mmol) was added dropwise at room temperature and the mixture was stirred overnight. The solvent was removed under vacuum, and the resulting white solid was washed with anhydrous n-hexane and dried under vacuum to give 1 g of **1** (yield = 73%). **1** was then stored in a glove box. The complex is soluble in polar solvents like acetone, THF, CH_3_CN, and slightly soluble in CH_2_Cl_2_ and CHCl_3_.

**1:** C_20_H_12_O_4_F_6_Zn. MW = 495 g·mol^−1^. ^1^H NMR (CDCl_3_, δ ppm): 7.99 (4H, d, *J* = 7.43 Hz, HC_ar_); 7.6 (2H, t, *J* = 7.36 Hz, HC_ar_); 7.48 (4H, t, *J* = 7.36 Hz, HC_ar_); 6.63 (2H, s, -CH-). ^19^F NMR (CDCl_3_, δ ppm): −75.55.

**[Zn(L^1^)_2_(H_2_O)_2_] (2)**

The same procedure used for the synthesis of compound **1** was repeated, except the reaction mixture was exposed to air immediately after the reaction was stopped. After solvent removal via rotary evaporation, a resulting white solid of **2** (2.2 g, 90%) was obtained and collected by filtration after being washed by n-hexane. The complex is soluble in polar solvents such as acetone, THF, CH_3_CN, and slightly soluble in CH_2_Cl_2_ and CHCl_3_. Colorless single crystals suitable for X-ray diffraction were obtained by slow evaporation from an CH_3_CN solution of **2**.

**2:** C_20_H_16_O_6_F_6_Zn. MW = 531 g·mol^−1 1^H NMR (CDCl_3_, δ ppm): 7.92 (4H, d, *J* = 8.16 Hz, HC_ar)_; 7.6 (2H, t, *J* = 7.05 Hz, HC_ar_); 7.48 (4H, t, *J* = 7.92 Hz, HC_ar_); 6.6 (2H, s, -CH-). ^19^F NMR (CDCl_3_, δ ppm): −75.58 ppm. FT-IR (cm^−1^): 3363 cm^−1^ (νO-H of H_2_O), 3070 cm^−1^ (νCsp^2^ - H), 1606 cm^−1^ (νC=O), 1573 cm^−1^ (νC=C), 1311 cm^−1^ (νC-O). ESI (*m*/*z*): [M + Na^+^]^+^ = 516.98. UV-vis (absolute EtOH): λ_1_max = 328 nm. Band gap = 3.78 eV.

**[Zn(L^1^)_2_(TMEDA)] (3)**

Volumes of 531 mg of [Zn(L^1^)_2_(H_2_O)_2_] **2** (1 mmol) and 0.18 mL of TMEDA (1.2 mmol) were mixed in 10 mL of THF and the mixture was stirred for 18 hrs at room temperature. Both THF and TMEDA were removed by rotary evaporator at 65 °C. The resulting white solid **3** (0.46 g, 80%) was washed with n-hexane (20 mL) and collected and dried on a Büchner. The complex is moderately soluble in highly polar solvents such as acetone and CH_3_CN but only slightly soluble in less polar solvents such as CH_2_Cl_2_, CHCl_3_, and others. Colorless single crystals suitable for X-ray diffraction were obtained by slow evaporation from a saturated CH_3_CN solution of **3**.

**3:** C_26_H_28_O_4_F_6_N_2_Zn. MW = 611 g·mol^−1^. ^1^H NMR ((CD_3_)_2_CO, δ ppm): 7.91 (4H, d, *J* = 7.21 Hz, HC_ar_); 7.50 (2H, t, *J* = 7.2 Hz, HC_ar_); 7.39 (4H, t, *J* = 7.52 Hz, HC_ar_); 6.28 (2H, s, -CH-); 2.85 (4H, s, -CH_2_N); 2.49 (12H, s, CH_3_N).^19^F NMR ((CD_3_)_2_CO, δ ppm): 100.89 ppm. FT-IR (cm^−1^): 2800–3070 cm^−1^ (νCsp^2^/Csp^3^ - H),1621 cm^−1^ (νC=O), 1573 cm^−1^ (νC=C), 1311 cm^−1^ (νC-O). ESI (*m*/*z*): ESI (*m*/*z*): [M + Na^+^]^+^ = 633.11. UV-vis (absolute EtOH): λ_1_max = 323 nm. Band gap = 3.84 eV.

**[Zn(L^1^)_2_(bipy)] (4)**

The same procedure used for the synthesis of compound **3** was followed to give **4** by mixing 531 mg of **2** (1 mmol) and 187.5 mg of bipy (1.2 mmol) in 10 mL THF. After stirring, the resulting white solid **4** (0.334 g, 50%) was obtained and washed with an EtOAc-n-hexane mixture (2:8) to remove the excess bipy. The complex is soluble in polar solvents like acetone, THF, CH_3_CN, and slightly soluble in CH_2_Cl_2_ and CHCl_3_. Colorless single crystals suitable for X-ray diffraction were obtained by slow evaporation from a saturated CH_3_CN solution of **4**.

**4:** C_30_H_20_O_4_F_6_N_2_Zn. MW = 651 g·mol^−1 1^H NMR ((CD_3_)_2_CO, δ ppm): 8.82 (2H, d, *J* = 4.36 Hz, HC_bipy_); 8.67 (2H, d, *J* = 8.24 Hz, HC_bipy_); 8.33 (2H, td, ^3^*J* = 1.4 Hz, ^4^*J* = 9.07 Hz, HC_bipy_); 7.85 (6H, m, HC_bipy_ + HC_ar_); 7.5 (2H, t, *J* = 7.34 Hz, HC_ar_); 7.39 (4H, t, *J* = 7.5 Hz, HC_ar_); 6.33 (2H, s, -CH-). ^19^F NMR ((CD_3_)_2_CO, δ ppm): 101.19 ppm. FT-IR (cm^−1^): 3029-3114 cm^−1^ (νCsp^2^/Csp^3^ - H), 1612 cm^−1^ (νC=O), 1577 cm^−1^ (νC=C), 1311 cm^−1^ (νC-O). ESI (*m*/*z*): [M + Na^+^]^+^ = 673.05. UV-vis (absolute EtOH): λ_1_max = 324 nm. Band gap = 3.83 eV.

**[Zn(L^1^)_2_(*o*-phen)] (5)**

The same procedure used for the synthesis of compound **4** was followed for **5** by mixing 531 mg of **2** (1 mmol) and 218 mg of *o*-phen.H_2_O (1.1 mmol) in 10 mL THF. Compound **5** (0.50 g, 74%) was collected after its impure form **5** was dissolved in CH_3_CN (50 mL: 10 mL for each 100 mg of product) to remove the excess of *o*-phen which precipitated. This is followed by washing with n-hexane (20 mL) and drying on a Büchner to obtain the pure complex. The complex is soluble in polar solvents like acetone, THF, CH_3_CN, and slightly soluble in CH_2_Cl_2_ and CHCl_3_. Colorless single crystals suitable for X-ray diffraction were obtained by slow evaporation from a saturated CH_3_CN solution of **5**.

**5**: C_32_H_20_O_4_F_6_N_2_Zn. MW = 675 g·mol^−1^. ^1^H NMR ((CD_3_)_2_CO, δ ppm): 9.13 (2H, dd, *J* = 4.16 Hz and *J* = 1.31 Hz, HC*_o_*_-phen_); 8.86 (2H, dd, *J* = 8.33 Hz and *J* = 1.34 Hz, HC*_o_*_-phen_); 8.23 (2H, dd, *J* = 8.22 Hz and *J* = 4.3 Hz, HC*_o_*_-phen_); 8.14 (2H, q, *J* = 8.2 Hz, HC*_o_*_-phen_); 7.89 (4H, d, *J* = 7.22 Hz, HC_ar_); 7.49 (2H, t, *J* = 7.3 Hz, HC_ar_); 7.4 (4H, t, *J* = 7.44 Hz, HC_ar_); 6.4 (2H, s, -CH-). ^19^F NMR ((CD_3_)_2_CO, δ ppm): 101.27 ppm. FT-IR (cm^−1^) 3025-3070 cm^−1^ (νCsp^2^/Csp^3^ - H), 1610 cm^−1^ (νC=O), 1575 cm^−1^ (νC=C), 1286 cm^−1^ (νC-O). ESI (*m*/*z*): [M + Na^+^]^+^ = 697.05. UV-vis (absolute EtOH): λ_1_max = 322 nm. Band gap = 3.85 eV.

## 4. Conclusions

This work addresses the synthesis and full characterization of a series of homoleptic and heteroleptic Zn(II) complexes bearing donor-acceptor β-diketonate ligands. The most significant outcome stems from ultrafast spectroscopic studies, which showed that coordination to zinc fundamentally redirects the photophysical pathway of the β-diketonate ligand. Unlike the free ligand, which relaxes rapidly to the ground state, the zinc-complexed form populates a long-lived triplet state. The observed stabilization of long-lived triplet states in these zinc β-diketonate complexes demonstrates that careful ligand design can control excited-state dynamics and reactive intermediate formation. The persistence of the triplet state, which can promote hydrogen abstraction from the solvent, suggests a photochemical decomposition pathway distinct from classical thermal mechanisms. This insight reveals a rational route to tailoring molecular precursors for targeted photochemical processes, where selective ligand loss or Zn-O bond activation may be triggered under UV excitation. Moreover, the minimal influence of the ancillary ligands on the excited-state kinetics highlights that the β-diketonate framework primarily governs the relaxation dynamics and photoreactivity. The correlation between ligand structure, photophysical behavior, and thermal stability thus provides a predictive framework for designing ZnO-relevant precursors and other functional materials. Future studies employing nonlinear photoexcitation can exploit these properties to achieve precise 3D nanostructure fabrication, controlled photodecomposition, and energy-directed material growth, emphasizing the potential of these complexes in advanced photonic and electronic applications.

## Figures and Tables

**Table 1:** Selected bond lengths (Å) and angles (°) of complexes [Zn(L^1^)_2_(H_2_O)_2_] (**2**), [Zn(L^1^)_2_(TMEDA)] (**3**), [Zn(L^1^)_2_(bipy)] (**4**), and [Zn(L^1^)_2_(*o*-phen)] (**5**). Standard deviations are in ().

Å	**2**	**3**	**4**	**5**
Zn-O1	2.0534 (10)	2.0661 (15)	2.1059 (12)	2.0714 (16)
Zn-O2	2.0534 (10)	2.0661 (15)	2.0579 (13)	2.0712 (17)
Zn-O3	2.0953 (10)	2.0856 (17)	2.0686 (12)	2.0667 (15)
Zn-O4	2.0953 (10)	2.0856 (17)	2.1251 (11)	2.1330 (15)
Zn-O5	2.1197 (11)	-	-	-
Zn-O6	2.1197 (11)	-	-	-
Zn-N1	-	2.1981 (19)	2.1190 (14)	2.1369 (18)
Zn-N2	-	2.1981 (19)	2.1228 (14)	2.1476 (19)
^o^	**2**	**3**	**4**	**5**
O1-Zn-O2	180.00 (5)	175.53 (7)	86.16 (5)	84.83 (6)
O3-Zn-O4	180.00 (5)	92.25 (10)	91.38 (5)	90.33 (6)
O3-Zn-N2	-	92.38 (7)	92.69 (5)	90.55 (7)
O4-Zn-N1	-	92.38 (7)	84.61 (5)	94.38 (6)

**Table 2:** Theoretical (m_residue_/m_complex_ × 100) values for ZnO and ZnF_2_ compared with experimental residues.

Complex	Theoretical % of Residues for ZnO	Theoretical % of Residues for ZnF_2_	Experimental % Residues at 600 °C
**1**	16	21	32
**2**	15	19	32
**3**	13	17	38
**4**	12	16	46
**5**	12	15	66

## Data Availability

Data are contained within the article and Supplementary Materials.

## Supplementary Materials

The following supporting data can be downloaded at https://www.mdpi.com/article/10.3390/molecules30224325/s1. Spectroscopic Characterizations: Complex **1**: NMR (CDCl_3_, 300 MHz, ^1^H, and ^19^F); complex **2**: NMR (CDCl_3_, 300 MHz, ^1^H, and ^19^F); complex **3**: NMR ((CD_3_)_2_CO, 300 MHz, ^1^H, and ^19^F); complex **4**: NMR ((CD_3_)_2_CO, 300 MHz, ^1^H, and ^19^F); complex **5**: NMR ((CD_3_)_2_CO, 300 MHz, ^1^H, and ^19^F). **Single-Crystal X-ray Characterizations of the zinc complexes**: Complex **2**; complex **3**; complex **4**; and complex **5**. Table S1. Crystallographic data of complexes [Zn(L^1^)_2_(H_2_O)_2_] (**2**), [Zn(L^1^)_2_(TMEDA)] (**3**), [Zn(L^1^)_2_(bipy)] (**4**), and [Zn(L^1^)_2_(*o*-phen)] (**5**). Table S2. Bond lengths and angles of complexes [Zn(L^1^)_2_(H_2_O)_2_] (**2**), [Zn(L^1^)_2_(TMEDA)] (**3**), [Zn(L^1^)_2_(bipy)] (**4**), and [Zn(L^1^)_2_(*o*-phen)] (**5**). **ATR-FTIR Spectra**: Figure S1. Infrared spectrum of ligand **L^1^H**; Figure S2. Infrared spectrum of complex **2**; Figure S3. Infrared spectrum of complex **3**; Figure S4. Infrared spectrum of complex **4**; and Figure S5. Infrared spectrum of complex **5**. Thermal Analysis**:** Figure S6. The thermal property (TGA) study of the ligand L^1^H and its corresponding complexes (1–5). **Differential Scanning Calorimetry thermograms:** Figure S7. DSC thermogram of complex **2:** first heating (black)-dehydration at 118 °C and 147 °C, melting at 167 °C; cooling (red); second heating (blue)-crystallization at 88 °C and 114 °C, melting at 162 °C. m = 7.13 mg; Figure S8. Differential Scanning Calorimetry (DSC) thermogram of complex **5**. The first heating (pink), cooling (green), and second heating (black) scans show no detectable thermal transitions. Sample mass: 6.4 mg. **ESI Mass Spectrometry**: Figure S9. Mass spectrometry of complex 2; Figure S10. Mass spectrometry of complex 3; Figure S11. Mass spectrometry of complex 4; Figure S12. Mass spectrometry of complex 5. **UV-Vis absorption spectroscopy**: Figure S13. The UV-vis study of the ligand (**L^1^H**) and its corresponding complexes (**2**–**5**). **Femtosecond transient absorption spectroscopy (TAS):** Figure S14. Transient absorption spectra of complex [Zn(L^1^)_2_(TMEDA)] (**3**) recorded in EtOH with a pump excitation λ_pump_ = 320 nm; Figure S15. Transient absorption spectra of compounds [Zn(L^1^)_2_(bipy)] (**4**) recorded in EtOH with a pump excitation λ_pump_ = 320 nm; Figure S16. Transient absorption spectra of compounds [Zn(L^1^)_2_(*o*-phen)] (**5**) recorded in EtOH with a pump excitation λ_pump_ = 320 nm. Crystal data were deposited in the CCDC with the numbers 2384613, 2384615, 2384616, and 2384614, for the complexes **2**, **3**, **4**, and **5**; **fs/ps Decay-Associated Spectra and kinetics:** Figure S17. Principal kinetic traces (left) and decay-associated spectra and their decay times (right) calculated from the TAS data of complex [Zn(L^1^)_2_(H_2_O)_2_] (**2**); Figure S18. Principal kinetic traces (left) and decay-associated spectra and their decay times (right) calculated from the TAS data of complex [Zn(L^1^)_2_(TMEDA)] (**3**); Figure S19. Principal kinetic traces (left) and decay-associated spectra and their decay times (right) calculated from the TAS data of complex [Zn(L^1^)_2_(bipy)] (**4**); Figure S20. Principal kinetic traces (left) and decay-associated spectra and their decay times (right) calculated from the TAS data of complex [Zn(L^1^)_2_(*o*-phen)] (**5**); Figure S21. Normalized decay-associated spectra of the long-living species observed in the TAS data of the complex **2**–**5**; and Table S3. The decay data for each complex are presented in Figure S21. Table S4. C,H,N elemental Analysis for complexes **2**–**5**.

## Abbreviations

The following abbreviations are used in this manuscript:

ESI	Electro-Spray Ionization
UV-Vis	Ultraviolet-Visible Spectroscopy
TGA	Thermogravimetric Analysis
ZnO	Zinc Oxide
TMEDA	N,N,N’,N’-Tetramethylethylenediamine
bipy	2,2′-Bipyridine
*o*-phen	1,10-Phenanthroline
CIF	Crystallographic Information File
LMCT	Ligand-to-Metal Charge Transfer
ESA	Excited-State Absorption
EtOH	Ethanol
CH_2_Cl_2_	Dichloromethane
CHCl_3_	Chloroform
THF	Tetrahydrofuran
CH_3_CN	Acetonitrile
H_2_O	Water
ZnEt_2_	Diethylzinc
**L^1^H**	4,4,4-Trifluoro-1-phenylbutane-1,3-dione
LB	Lewis Base
DBM	Dibenzoylmethane
