# Supplementary material for: Zinc β-Diketonates with Donor-Acceptor Ligands: Synthesis and Comprehensive Structural, Thermal, and Photophysical Characterization
Source: Molecules. 2025 Nov 7;30(22):4325. doi: 10.3390/molecules30224325 (PMC12654558; doi:10.3390/molecules30224325)
Supplement: Supplementary file 1 [file molecules-30-04325-s001.zip › molecules-3931125-supplementary.pdf]

Supporting infromation

Zinc β-Diketonates with Donor-Acceptor Ligands: Synthesis and Comprehensive Structural, Thermal, and Photophysical Characterization

Ahmad Daher [1], Manjiri Choudhari [2], Thomas Roland [2], Vincent De Waele [2] and Stéphane Daniele [1,*]

[1] Catalyse, Polymérisation, Procédés et Matériaux (CP2M), Chimie École Supérieure de Chimie Physique Électronique de Lyon (CPE Lyon), CNRS UMR 5128, Université de Lyon, F-69100 Villeurbanne, France; ahmad.daher@univ-lyon1.fr

[2] Univ. Lille, CNRS, UMR 8516, LASIRE-Laboratoire de Spectroscopie pour les Interactions, la Réactivité et l'Environnement, 59000 Lille, France; choudhari.manjiri@univ-lille.fr (M.C.); thomas.roland@univ-lille.fr (T.R.); vincent.de-waele@univ-lille.fr (V.D.W.)

* Correspondence: stephane.daniele@univ-lyon1.fr

Table Of Contents

Supporting infromation 1
Experimental part 3
Spectroscopic Characterizations : Copy of NMR spectrum 3
Complex **1**: NMR ($CDCl_3$, 300 MHz, ^1H and ^{19}F) 3
Complex **2**: NMR ($CDCl_3$, 300 MHz, ^1H and ^{19}F) 4
Complex **3**: NMR ($(CD_3)_2CO$, 300 MHz, ^1H and ^{19}F) 6
Complex **4**: NMR ($(CD_3)_2CO$, 300 MHz, ^1H and ^{19}F) 7
Complex **5**: NMR ($(CD_3)_2CO$, 300 MHz, ^1H and ^{19}F) 8
Single-Crystal X-ray Characterizations of the zinc complexes 9
Complex **2** 9
Complex **3** 11
Complex **4** 13
Complex **5** 15
Citations 16
Optical properties 18
ATR-FTIR Spectra 18
L^1H 18
Complex **2** 19
Complex **3** 19
Complex **4** 20
Complex **5** 20
Thermal analysis (mass loss (%) vs. temperature (°C)), under N_2 atmosphere, sample mass = 10–12 mg 21

Differential Scanning Calorimetry thermograms of complexes 2 and 5 (top to bottom: first heating, cooling, second heating) recorded from 25 to 220 °C under N_2 (30 mL min^{-1}) at 10 °C min^{-1} using a 40 μL aluminum pan 21

Complex **2** 21

Complex **5** 22

ESI Mass Spectrometry (mass range 50–1500 m/z): nebulizer pressure = 0.3 bar, source temperature = 200 °C, capillary voltage = 2500 V, dry gas flow = 4.0 L min^{-1}, end plate offset = –500 V 22

Complex **2** 22

Complex **3** 23

Complex **4** 23

Complex **5** 24

UV–Vis (Absorbance vs. wavelength (nm)), concentration =10^{-5} M, quartz cell, path length = 1 cm. 24

Femtosecond transient absorption spectroscopy (TAS) 25

TAS Spectra 25

Complex **3** 25

Complex **4** 25

Complex **5** 26

fs/ ps Decays Associated Spectra and kinetics 27

Complex **2** 27

Complex **3** 28

Complex **4** 28

Complex **5** 29

Experimental part

Spectroscopic Characterizations : Copy of NMR spectrum

Complex **1**: NMR ($CDCl_3$, 300 MHz, ^1H and ^{19}F)

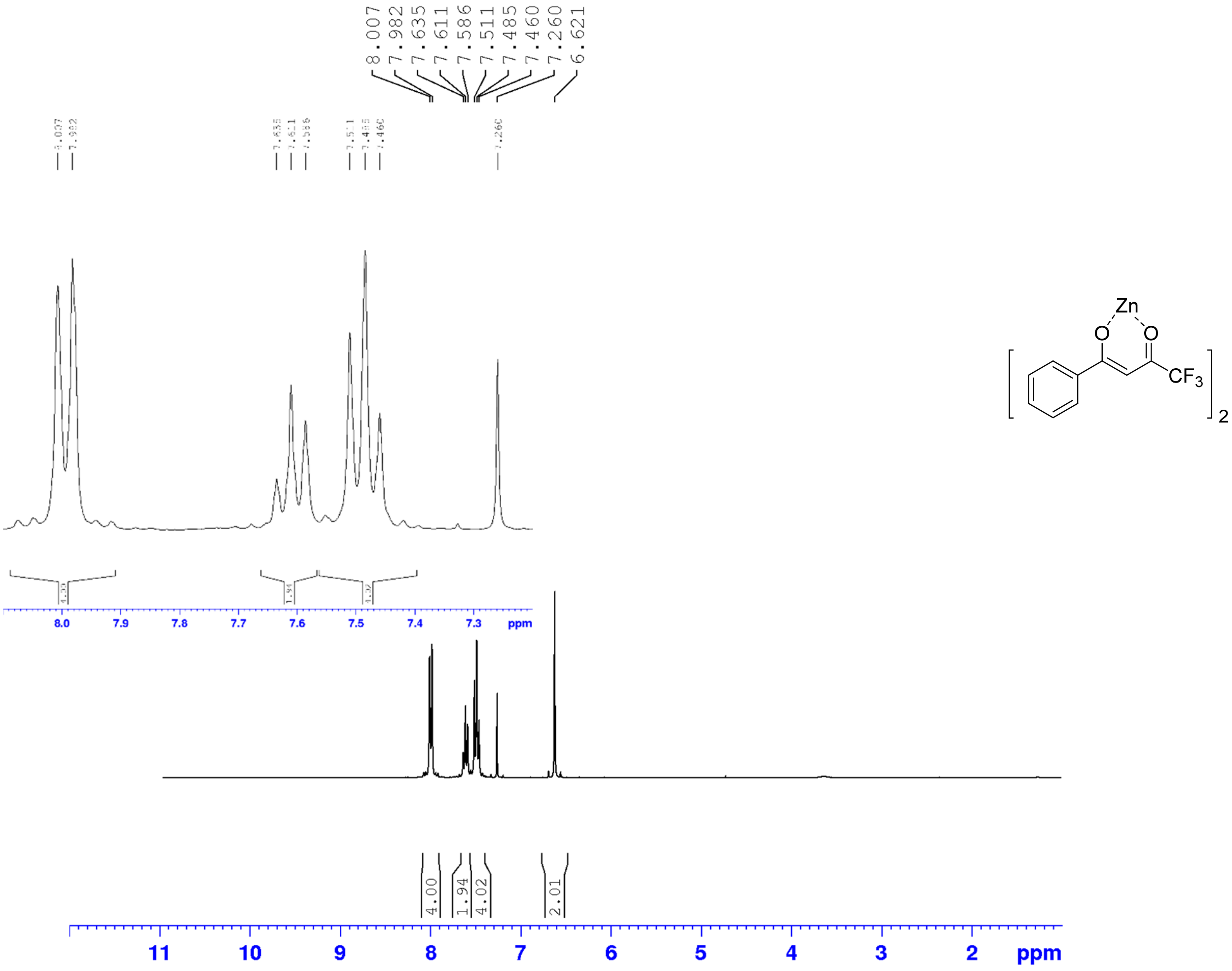

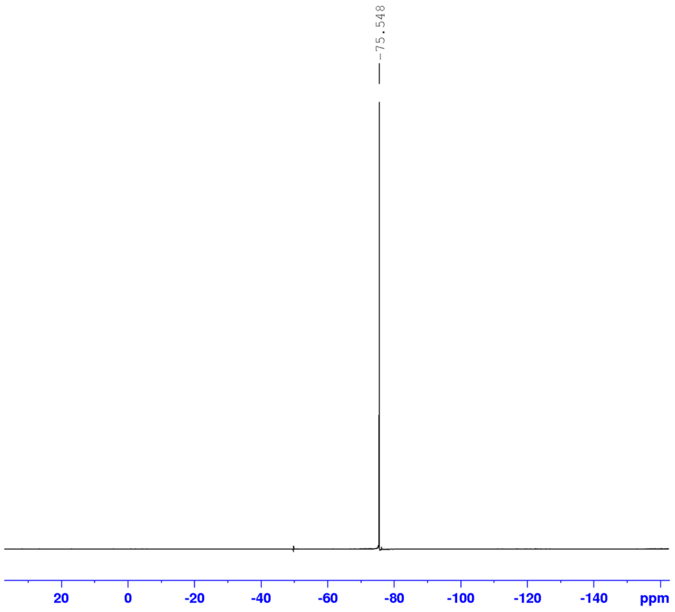

Complex **2**: NMR ($CDCl_3$, 300 MHz, ^1H and ^{19}F)

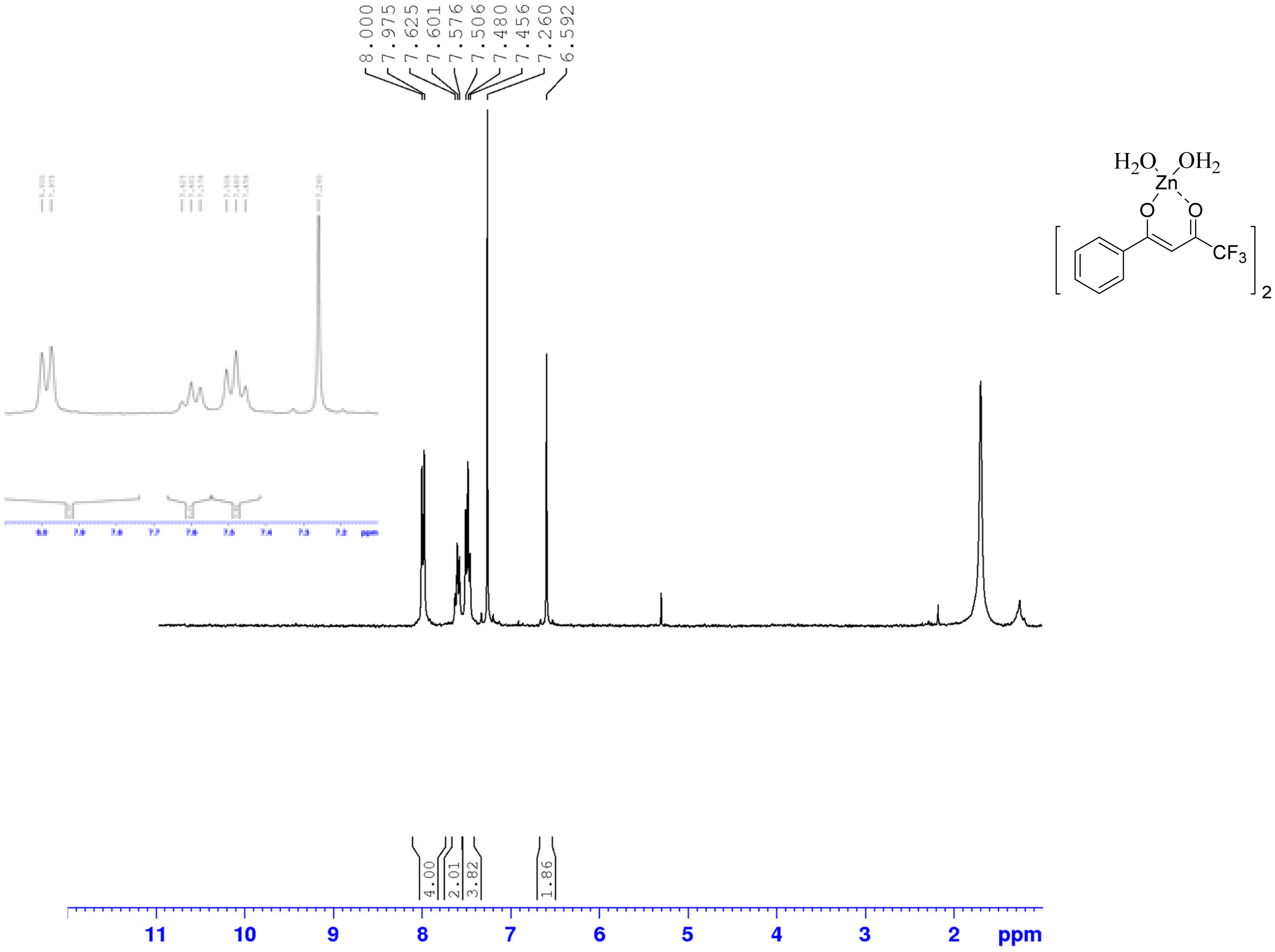

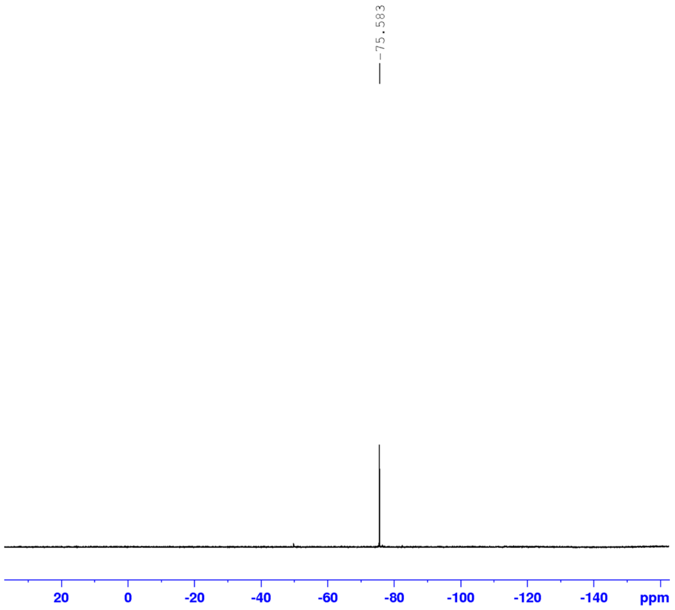
-75.583
20
0
-20
-40
-60
-80
-100
-120
-140
ppm

Complex **3**: NMR ($(CD_3)_2CO$, 300 MHz, ^{1}H and ^{19}F)

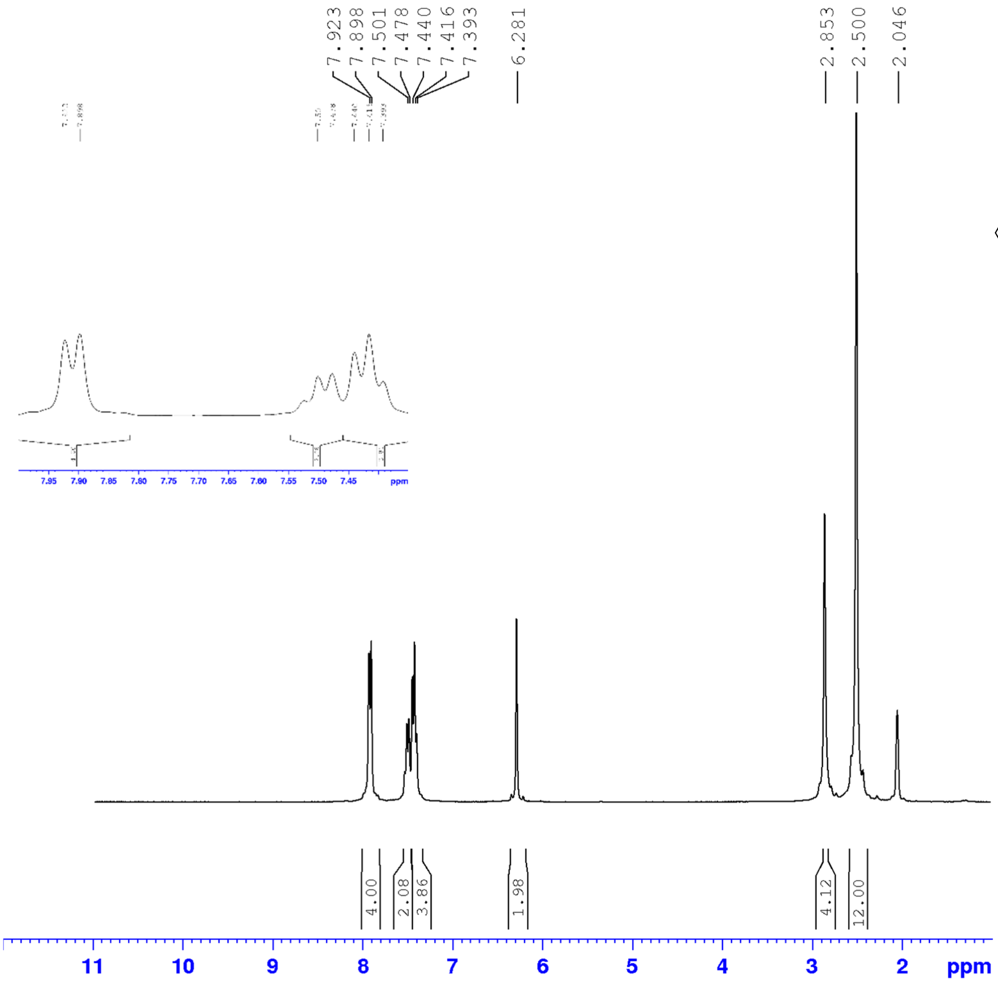

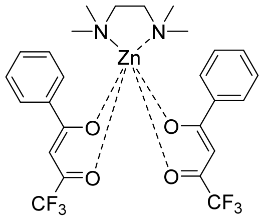

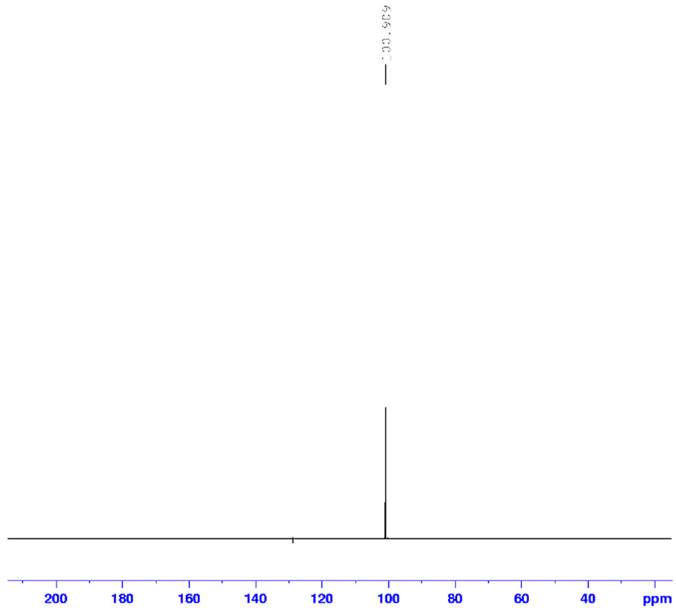

Complex **4**: NMR ($(CD_3)_2CO$, 300 MHz, ^1H and ^{19}F)

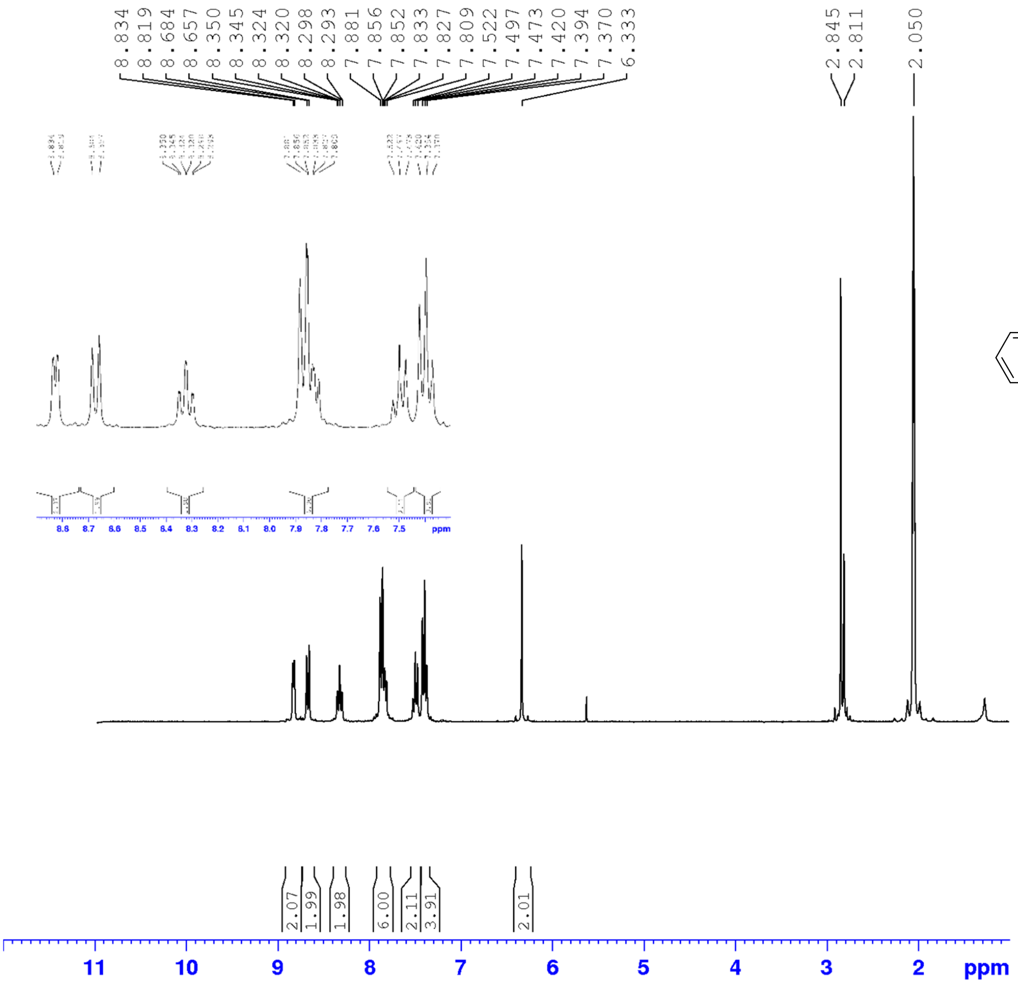

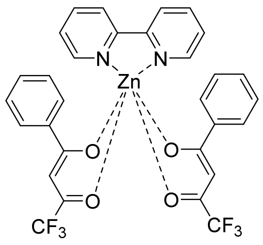

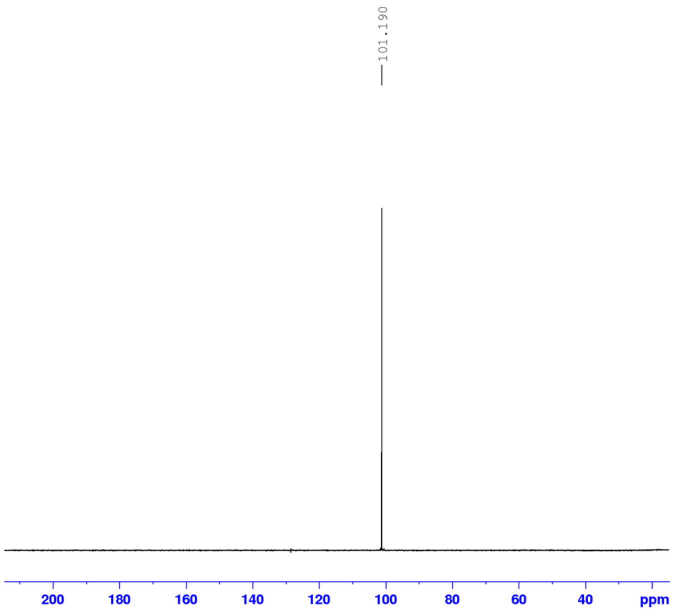

Complex **5**: NMR ($(CD_3)_2CO$, 300 MHz, ^1H and ^{19}F)

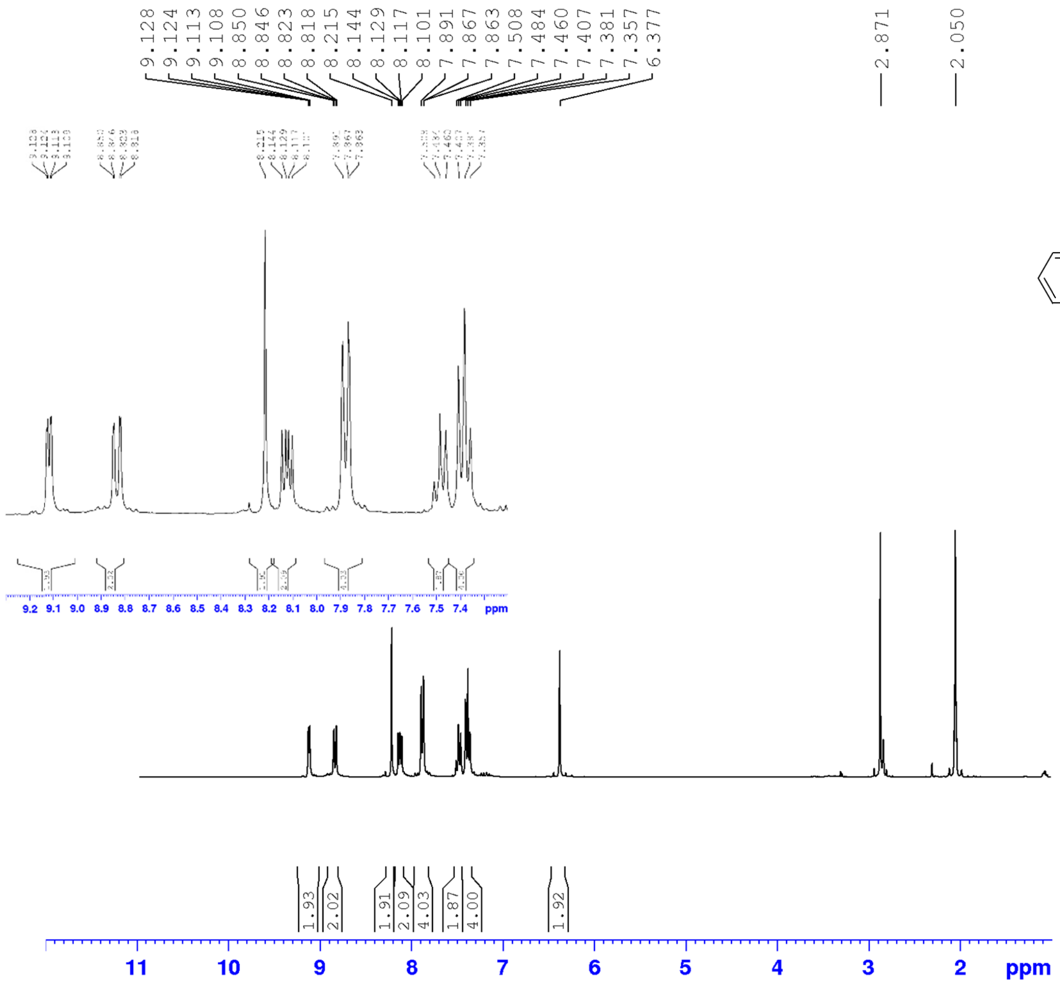

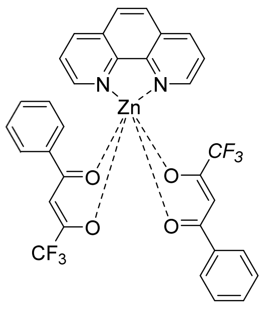

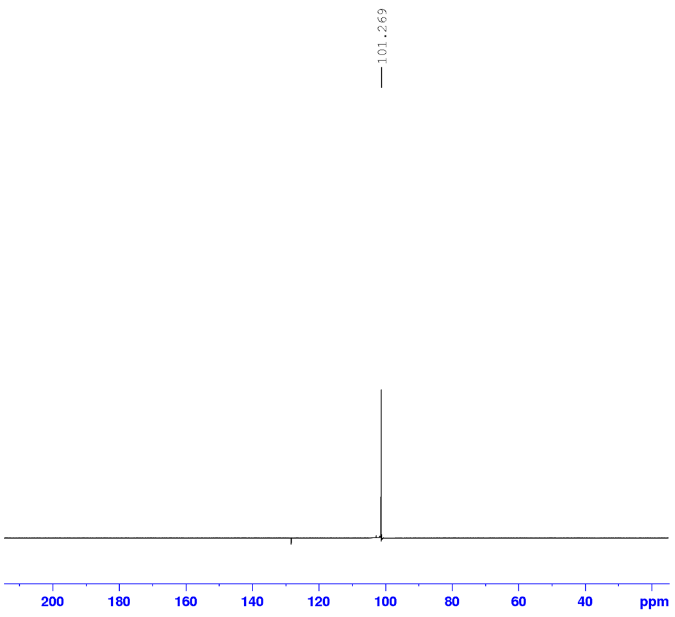

Single-Crystal X-ray Characterizations of the zinc complexes

Complex **2**

R_1=2.61%

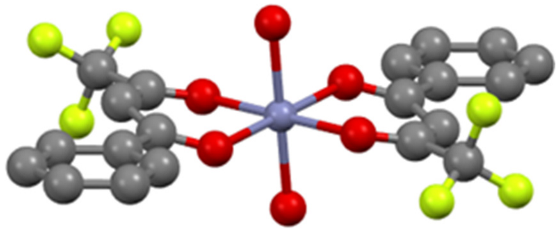

Experimental. Single colourless plate-shaped crystals of complex **2** were used as supplied. A suitable crystal with dimensions 0.28 × 0.14 × 0.02 mm³ was selected and mounted on a XtaLAB Synergy, Dualflex, HyPix-Arc 100 diffractometer. The crystal was kept at a steady T = 100.0(2) K during data collection. The structure was solved with the ShelXT 2018/2 (Sheldrick, 2018) solution program using dual methods and by using Olex2 1.5-ac6-015 (Dolomanov et al., 2009) as the graphical interface. The model was refined with ShelXL 2018/3 (Sheldrick, 2015) using full matrix least squares minimisation on $\boldsymbol{F}^2$.

Crystal Data. $C_{20}H_{16}F_6O_6Zn$, M_r = 531.70, triclinic, P-1 (No. 2), a = 4.80150(10) Å, b = 9.5125(2) Å, c = 11.5291(2) Å, α = 75.925(2)°, β = 78.557(2)°, γ = 80.2440(10)°, V = 496.536(18) Å³, T = 100.0(2) K, Z = 1, Z' = 0.5, μ(Mo K_α) = 1.330, 21817 reflections measured, 2677 unique (R_{int} = 0.0317) which were used in all calculations. The final wR_2 was 0.0619 (all data) and R_1 was 0.0261 (I≥2 σ(I)).

**Complex**	**2**
Formula	$C_{20}H_{16}F_6O_6Zn$
$D_{calc.}$/ g cm^{-3}	1.778
μ/mm^{-1}	1.330
Formula Weight	531.70
Colour	colourless
Shape	plate-shaped
Size/mm³	0.28×0.14×0.02
T/K	100.0(2)
Crystal System	triclinic
Space Group	P-1
a/Å	4.80150(10)
b/Å	9.5125(2)
c/Å	11.5291(2)
α/°	75.925(2)
β/°	78.557(2)
γ/°	80.2440(10)
V/Å³	496.536(18)
Z	1
Z'	0.5
Wavelength/Å	0.71073
Radiation type	Mo K_α
Θ_{min}/°	2.565
Θ_{max}/°	30.286
Measured Refl's.	21817
Indep't Refl's	2677
Refl's I≥2 σ(I)	2483
R_{int}	0.0317
Parameters	159
Restraints	0
Largest Peak	0.407
Deepest Hole	-0.388
GooF	1.086
wR_2 (all data)	0.0619
wR_2	0.0608
R_1 (all data)	0.0295
R_1	0.0261

Structure Quality Indicators

**Reflections:**	d min (MoKα) 2Θ=60.6° 0.70	I/σ(I) 49.3	Rint m=8.16 3.17%	Full 50.5° 90% to 60.6° 99.8
**Refinement:**	Shift -0.001	Max Peak 0.4	Min Peak -0.4	GooF 1.086

A colourless plate-shaped-shaped crystal with dimensions 0.28 × 0.14 × 0.02 mm^3 was mounted. Data were collected using a XtaLAB Synergy, Dualflex, HyPix-Arc 100 diffractometer operating at T = 100.0(2) K.

Data were measured using ω scans with Mo K_α radiation. The diffraction pattern was indexed and the total number of runs and images was based on the strategy calculation from the program CrysAlisPro system (CCD 43.105a 64-bit (release 11-01-2024)). The maximum resolution that was achieved was Θ = 30.286° (0.70 Å).

The unit cell was refined using CrysAlisPro 1.171.43.105a (Rigaku OD, 2024) on 16026 reflections, 73% of the observed reflections.

Data reduction, scaling and absorption corrections were performed using CrysAlisPro 1.171.43.105a (Rigaku OD, 2024). The final completeness is 99.80 % out to 30.286° in Θ. A multi-scan absorption correction was performed using CrysAlisPro 1.171.43.105a (Rigaku Oxford Diffraction, 2024) Empirical absorption correction using spherical harmonics, implemented in SCALE3 ABSPACK scaling algorithm.. The absorption coefficient μ of this material is 1.330 mm^{-1} at this wavelength (λ = 0.71073Å) and the minimum and maximum transmissions are 0.959 and 1.000.

The structure was solved and the space group P-1 (# 2) determined by the ShelXT 2018/2 (Sheldrick, 2018) structure solution program using using dual methods and refined by full matrix least squares minimisation on $\boldsymbol{F}^2$ using version 2018/3 of ShelXL 2018/3 (Sheldrick, 2015). All non-hydrogen atoms were refined anisotropically. Hydrogen atom positions were calculated geometrically and refined using the riding model. Most hydrogen atom positions were calculated geometrically and refined using the riding model, but some hydrogen atoms were refined freely.

_exptl_absorpt_process_details: CrysAlisPro 1.171.43.105a (Rigaku Oxford Diffraction, 2024) using spherical harmonics,implemented in SCALE3 ABSPACK scaling algorithm.

The value of Z' is 0.5. This means that only half of the formula unit is present in the asymmetric unit, with the other half consisting of symmetry equivalent atoms. The moiety formula is $C_{20}H_{16}F_6O_6Zn$.

Complex **3**

R_1=2.97%

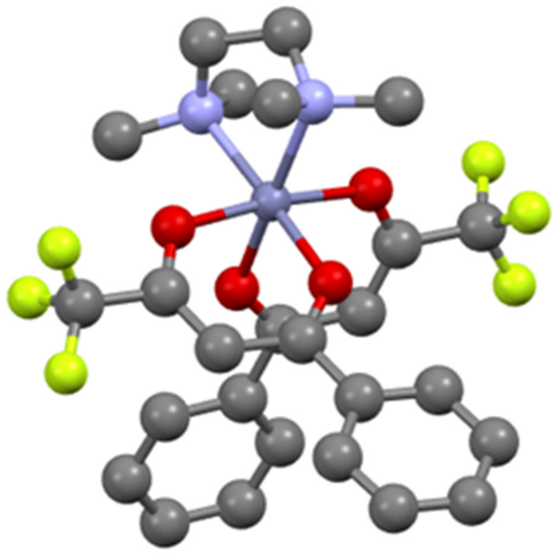

Experimental. Single colourless needle-shaped crystals of **complex 3** were used as supplied. A suitable crystal with dimensions 0.40 × 0.12 × 0.11 mm³ was selected and mounted on a XtaLAB Synergy, Dualflex, HyPix-Arc 100 diffractometer. The crystal was kept at a steady T = 100.0(3) K during data collection. The structure was solved with the ShelXT (Sheldrick, 2015) solution program using dual methods and by using Olex2 1.5-ac6-020 (Dolomanov et al., 2009) as the graphical interface. The model was refined with ShelXL 2018/3 (Sheldrick, 2015) using full matrix least squares minimisation on F^2.

Crystal Data. $C_{26}H_{28}F_6N_2O_4Zn$, M_r = 611.87, orthorhombic, $P2_12_12$ (No. 18), a = 8.53980(10) Å, b = 18.7656(2) Å, c = 17.20870(10) Å, $\alpha = \beta = \gamma$ = 90°, V = 2757.77(5) Å³, T = 100.0(3) K, Z = 4, Z' = 1, μ(Mo K_α) = 0.965, 184638 reflections measured, 8024 unique (R_{int} = 0.0464) which were used in all calculations. The final wR_2 was 0.0736 (all data) and R_1 was 0.0297 (I≥2 σ(I)).

**Complex**	**3**
Formula	$C_{26}H_{28}F_6N_2O_4Zn$
$D_{calc.}$/ g cm^{-3}	1.474
μ/mm^{-1}	0.965
Formula Weight	611.87
Colour	colourless
Shape	needle-shaped
Size/mm³	0.40×0.12×0.11
T/K	100.0(3)
Crystal System	orthorhombic
Flack Parameter	0.498(10)
Hooft Parameter	0.0080(15)
Space Group	$P2_12_12$
a/Å	8.53980(10)
b/Å	18.7656(2)
c/Å	17.20870(10)
α/°	90
β/°	90
γ/°	90
V/Å³	2757.77(5)
Z	4
Z'	1
Wavelength/Å	0.71073
Radiation type	Mo K_α
Θ_{min}/°	2.367
Θ_{max}/°	30.946
Measured Refl's.	184638
Indep't Refl's	8024
Refl's I≥2 σ(I)	7307
R_{int}	0.0464
Parameters	358
Restraints	0
Largest Peak	1.476
Deepest Hole	-0.410
GooF	1.048
wR_2 (all data)	0.0736
wR_2	0.0716
R_1 (all data)	0.0358
R_1	0.0297

Structure Quality Indicators

**Reflections:**	d min (MoKα) 2Θ=61.9° 0.69	I/σ(I) 58.4	Rint m=23.04 4.64%	Full 50.5° 94% to 61.9° 99.9
**Refinement:**	Shift 0.001	Max Peak 1.5	Min Peak -0.4	GooF 1.048

A colourless needle-shaped-shaped crystal with dimensions 0.40 × 0.12 × 0.11 mm^3 was mounted. Data were collected using a XtaLAB Synergy, Dualflex, HyPix-Arc 100 diffractometer operating at T = 100.0(3) K.

Data were measured using ω scans with Mo K_α radiation. The diffraction pattern was indexed and the total number of runs and images was based on the strategy calculation from the program CrysAlisPro system (CCD 43.123a 64-bit (release 20-05-2024)). The maximum resolution that was achieved was Θ = 30.946° (0.69 Å).

The unit cell was refined using CrysAlisPro 1.171.43.123a (Rigaku OD, 2024) on 103258 reflections, 56% of the observed reflections.

Data reduction, scaling and absorption corrections were performed using CrysAlisPro 1.171.43.123a (Rigaku OD, 2024). The final completeness is 99.90 % out to 30.946° in Θ. A multi-scan absorption correction was performed using CrysAlisPro 1.171.43.123a (Rigaku Oxford Diffraction, 2024) Empirical absorption correction using spherical harmonics, implemented in SCALE3 ABSPACK scaling algorithm.. The absorption coefficient μ of this material is 0.965 mm^{-1} at this wavelength (λ = 0.71073Å) and the minimum and maximum transmissions are 0.810 and 1.000.

The structure was solved and the space group $P2_12_12$ (# 18) determined by the ShelXT (Sheldrick, 2015) structure solution program using using dual methods and refined by full matrix least squares minimisation on $\boldsymbol{F}^2$ using version 2018/3 of ShelXL 2018/3 (Sheldrick, 2015). All non-hydrogen atoms were refined anisotropically. Hydrogen atom positions were calculated geometrically and refined using the riding model. Hydrogen atom positions were calculated geometrically and refined using the riding model.

_refine_special_details: Refined as a 2-component inversion twin.

_exptl_absorpt_process_details: CrysAlisPro 1.171.43.123a (Rigaku Oxford Diffraction, 2024) using spherical harmonics,implemented in SCALE3 ABSPACK scaling algorithm.

There is a single formula unit in the asymmetric unit, which is represented by the reported sum formula. In other words: Z is 4 and Z' is 1. The moiety formula is $C_{26}H_{28}F_6N_2O_4Zn$.

The Flack parameter was refined to 0.498(10). Determination of absolute structure using Bayesian statistics on Bijvoet differences using the Olex2 results in 0.0080(15). This structure is in chiral space group, but there are no chiral atoms. Note: The Flack parameter is used to determine chirality of the crystal studied, the value should be near 0, a value of 1 means that the stereochemistry is wrong and the model should be inverted. A value of 0.5 means that the crystal consists of a racemic mixture of the two enantiomers.

Complex **4**

R_1=3.57%

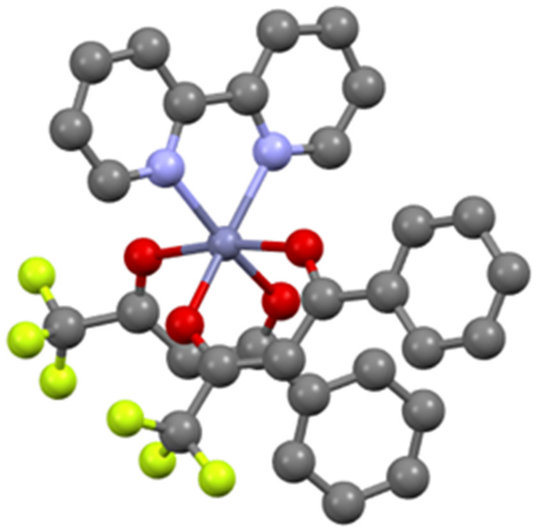

**Complex**	**4**
Formula	$C_{30}H_{20}F_6N_2O_4Zn$
$D_{calc.}$/ g cm^{-3}	1.545
μ/mm^{-1}	0.956
Formula Weight	651.85
Colour	colourless
Shape	block-shaped
Size/mm^3	0.30×0.26×0.20
T/K	100.00(10)
Crystal System	triclinic
Space Group	*P*-1
a/Å	8.5766(2)
b/Å	10.8852(2)
c/Å	15.7069(3)
α/°	90.703(2)
β/°	92.936(2)
γ/°	106.838(2)
V/Å^3	1401.09(5)
Z	2
Z'	1
Wavelength/Å	0.71073
Radiation type	Mo K_α
Θ_{min}/°	2.317
Θ_{max}/°	30.402
Measured Refl's.	62557
Indep't Refl's	7525
Refl's I≥2 σ(I)	6597
R_{int}	0.0430
Parameters	388
Restraints	0
Largest Peak	1.091
Deepest Hole	-0.693
GooF	1.076
wR_2 (all data)	0.0902
wR_2	0.0871
R_1 (all data)	0.0423
R_1	0.0357

Experimental. Single colourless block-shaped crystals of **complex 4** were used as supplied. A suitable crystal with dimensions 0.30 × 0.26 × 0.20 mm^3 was selected and mounted on a MITIGEN holder in perfluoroether oil on a XtaLAB Synergy, Dualflex, HyPix-Arc 100 diffractometer. The crystal was kept at a steady T = 100.00(10) K during data collection. The structure was solved with the ShelXT 2018/2 (Sheldrick, 2018) solution program using dual methods and by using Olex2 1.5-ac6-015 (Dolomanov et al., 2009) as the graphical interface. The model was refined with ShelXL 2018/3 (Sheldrick, 2015) using full matrix least squares minimisation on $\boldsymbol{F^2}$.

Crystal Data. $C_{30}H_{20}F_6N_2O_4Zn$, M_r = 651.85, triclinic, *P*-1 (No. 2), a = 8.5766(2) Å, b = 10.8852(2) Å, c = 15.7069(3) Å, α = 90.703(2)°, β = 92.936(2)°, γ = 106.838(2)°, V = 1401.09(5) Å^3, T = 100.00(10) K, Z = 2, Z' = 1, μ(Mo K_α) = 0.956, 62557 reflections measured, 7525 unique (R_{int} = 0.0430) which were used in all calculations. The final wR_2 was 0.0902 (all data) and R_1 was 0.0357 (I≥2 σ(I)).

Structure Quality Indicators

**Reflections:**	d min (MoKα) 2Θ=60.8° 0.70	I/σ(I) 39.2	Rint m=8.31 4.30%	Full 50.5° 89% to 60.8° 100
**Refinement:**	Shift -0.001	Max Peak 1.1	Min Peak -0.7	GooF 1.076

A colourless block-shaped-shaped crystal with dimensions 0.30 × 0.26 × 0.20 mm^3 was mounted on a MITIGEN holder in perfluoroether oil. Data were collected using a XtaLAB Synergy, Dualflex, HyPix-Arc 100 diffractometer operating at T = 100.00(10) K.

Data were measured using ω scans with Mo K_α radiation. The diffraction pattern was indexed and the total number of runs and images was based on the strategy calculation from the program CrysAlisPro system (CCD 43.120a 64-bit (release 16-04-2024)). The maximum resolution that was achieved was Θ = 30.402° (0.70 Å).

The unit cell was refined using CrysAlisPro 1.171.43.105a (Rigaku OD, 2024) on 40384 reflections, 65% of the observed reflections.

Data reduction, scaling and absorption corrections were performed using CrysAlisPro 1.171.43.105a (Rigaku OD, 2024). The final completeness is 100.00 % out to 30.402° in Θ. A gaussian absorption correction was performed using CrysAlisPro 1.171.43.105a (Rigaku Oxford Diffraction, 2024) Numerical absorption correction based on gaussian integration over a multifaceted crystal model Empirical absorption correction using spherical harmonics, implemented in SCALE3 ABSPACK scaling algorithm.. The absorption coefficient μ of this material is 0.956 mm^{-1} at this wavelength (λ = 0.71073Å) and the minimum and maximum transmissions are 0.200 and 1.000.

The structure was solved and the space group *P*-1 (# 2) determined by the ShelXT 2018/2 (Sheldrick, 2018) structure solution program using using dual methods and refined by full matrix least squares minimisation on $\boldsymbol{F}^2$ using version 2018/3 of ShelXL 2018/3 (Sheldrick, 2015). All non-hydrogen atoms were refined anisotropically. Hydrogen atom positions were calculated geometrically and refined using the riding model. Hydrogen atom positions were calculated geometrically and refined using the riding model.

_exptl_absorpt_process_details: CrysAlisPro 1.171.43.105a (Rigaku Oxford Diffraction, 2024)Numerical absorption correction based on gaussian integration overa multifaceted crystal modelEmpirical absorption correction using spherical harmonics,implemented in SCALE3 ABSPACK scaling algorithm.

There is a single formula unit in the asymmetric unit, which is represented by the reported sum formula. In other words: Z is 2 and Z' is 1. The moiety formula is C30 H20 F6 N2 O4 Zn.

Complex **5**

R_1=4.10%

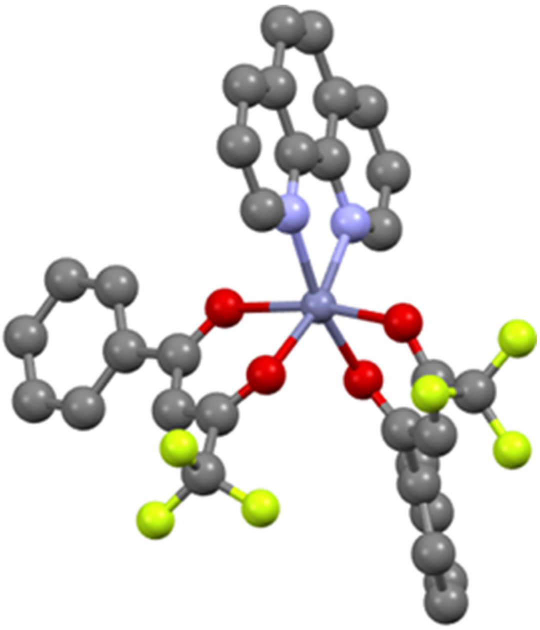

**Complex**	**5**
Formula	$C_{32}H_{20}N_2O_4F_6Zn$
$D_{calc.}$/ g cm^{-3}	1.564
μ/mm^{-1}	1.913
Formula Weight	675.87
Colour	colourless
Shape	block-shaped
Size/mm^3	0.11×0.07×0.02
T/K	200(140)
Crystal System	triclinic
Space Group	P-1
a/Å	8.81410(10)
b/Å	10.8811(2)
c/Å	15.5729(3)
α/°	94.330(2)
β/°	94.6610(10)
γ/°	104.339(2)
V/Å^3	1435.15(4)
Z	2
Z'	1
Wavelength/Å	1.54184
Radiation type	Cu K_α
Θ_{min}/°	2.861
Θ_{max}/°	77.775
Measured Refl's.	38401
Indep't Refl's	5687
Refl's I≥2 σ(I)	5220
R_{int}	0.0359
Parameters	406
Restraints	0
Largest Peak	0.963
Deepest Hole	-0.550
GooF	1.041
wR_2 (all data)	0.1105
wR_2	0.1083
R_1 (all data)	0.0444
R_1	0.0410

Experimental. Single colourless block-shaped crystals of **complex 5** were used as supplied. A suitable crystal with dimensions 0.11 × 0.07 × 0.02 mm^3 was selected and mounted on a MITIGEN holder in perfluoroether oil on a XtaLAB Synergy, Dualflex, HyPix-Arc 100 diffractometer. The crystal was kept at a steady T = 200(140) K during data collection. The structure was solved with the ShelXT 2018/2 (Sheldrick, 2018) solution program using dual methods and by using Olex2 1.5-ac6-020 (Dolomanov et al., 2009) as the graphical interface. The model was refined with ShelXL 2018/3 (Sheldrick, 2015) using full matrix least squares minimisation on $\boldsymbol{F}^2$.

Crystal Data. $C_{32}H_{20}N_2O_4F_6Zn$, M_r = 675.87, triclinic, P-1 (No. 2), a = 8.81410(10) Å, b = 10.8811(2) Å, c = 15.5729(3) Å, α = 94.330(2)°, β = 94.6610(10)°, γ = 104.339(2)°, V = 1435.15(4) Å^3, T = 200(140) K, Z = 2, Z' = 1, μ(Cu K_α) = 1.913, 38401 reflections measured, 5687 unique (R_{int} = 0.0359) which were used in all calculations. The final wR_2 was 0.1105 (all data) and R_1 was 0.0410 (I≥2 σ(I)).

Structure Quality Indicators

**Reflections:**	d min (CuKα) 2Θ=155.5° 0.79	I/σ(I) 49.2	Rint m=6.75 3.59%	Full 135.4° 93% to 155.5° 99.1
**Refinement:**	Shift 0.001	Max Peak 1.0	Min Peak -0.6	GooF 1.041

A colourless block-shaped-shaped crystal with dimensions 0.11 × 0.07 × 0.02 mm^3 was mounted on a MITIGEN holder in perfluoroether oil. Data were collected using a XtaLAB Synergy, Dualflex, HyPix-Arc 100 diffractometer operating at T = 200(140) K.

Data were measured using ω scans with Cu K_α radiation. The diffraction pattern was indexed and the total number of runs and images was based on the strategy calculation from the program CrysAlisPro system (CCD 43.125a 64-bit (release 04-06-2024)). The maximum resolution that was achieved was Θ = 77.775° (0.79 Å).

The unit cell was refined using CrysAlisPro 1.171.43.124a **[1]** on 20801 reflections, 54% of the observed reflections.

Data reduction, scaling and absorption corrections were performed using CrysAlisPro 1.171.43.124a **[1]**. The final completeness is 99.10 % out to 77.775° in Θ. A multi-scan absorption correction was performed using CrysAlisPro 1.171.43.124a **[1].** Empirical absorption correction using spherical harmonics, implemented in SCALE3 ABSPACK scaling algorithm.. The absorption coefficient μ of this material is 1.913 mm^{-1} at this wavelength (λ = 1.54184Å) and the minimum and maximum transmissions are 0.781 and 1.000.

The structure was solved and the space group P-1 (# 2) determined by the ShelXT 2018/2 **[2]** structure solution program using using dual methods and refined by full matrix least squares minimisation on $\boldsymbol{F}^2$ using version 2018/3 of ShelXL 2018/3 **[3]**. All non-hydrogen atoms were refined anisotropically **[4]**. Hydrogen atom positions were calculated geometrically and refined using the riding model. Hydrogen atom positions were calculated geometrically and refined using the riding model **[4]**.

_exptl_absorpt_process_details: CrysAlisPro 1.171.43.124a **[1]** using spherical harmonics,implemented in SCALE3 ABSPACK scaling algorithm.

There is a single formula unit in the asymmetric unit, which is represented by the reported sum formula. In other words: Z is 2 and Z' is 1. The moiety formula is C32 H20 F6 N2 O4 Zn.

Citations

[1] *CrysAlisPro*, Rigaku Oxford Diffraction (ROD) Ltd., Yarnton, Oxfordshire, England, (2024).
[2] Sheldrick, G.M., ShelXT-Integrated space-group and crystal-structure determination, *Acta Cryst.*, (2015), **A71**, 3-8.

[3] Sheldrick, G.M., Crystal structure refinement with ShelXL, *Acta Cryst.*, (2015), **C71**, 3-8.

[4] O.V. Dolomanov and L.J. Bourhis and R.J. Gildea and J.A.K. Howard and H. Puschmann, Olex2: A complete structure solution, refinement and analysis program, *J. Appl. Cryst.*, (2009), **42**, 339-341.

Table S1. Crystallographic data of complexes [Zn(L^1)$_2$(H$_2$O)$_2$] **(2)**, [Zn(L^1)$_2$(TMEDA)] **(3)**, [Zn(L^1)$_2$(bipy)] **(4)** and [Zn(L^1)$_2$(*o*-phen)] **(5)**

Complex	**2**	**3**	**4**	**5**
Formula	$C_{20}H_{16}O_6F_6Zn$	$C_{26}H_{28}N_2O_4F_6Zn$	$C_{30}H_{20}N_2O_4F_6Zn$	$C_{32}H_{20}N_2O_4F_6Zn$
M. W.	531.70	611.87	651.85	675.87
C. S.	Triclinic	Orthohombic	Triclinic	Triclinic
S. G.	P-1	$P2_12_12$	P-1	P-1
a (Å)	4.80150(10)	8.53980(10)	8.5766(2)	8.81410(10)
b (Å)	9.5125(2)	18.7656(2)	10.8852(2)	10.8811(2)
c (Å)	11.5291(2)	17.20870(10)	15.7069(3)	15.5729(3)
α (°)	75.925(2)	90	90.703(2)	94.330(2)
β (°)	78.557(2)	90	92.936(2)	94.6610(10)
γ (°)	80.2440(10)	90	106.838(2)	104.339(2)
V (Å^3)	496.536(18)	2757.77(5)	1401.09(5)	1435.15(4)
Z	1	4	4	2
p (g.cm^{-3})	1.778	1.474	1.545	1.564
μ (mm^{-1})	1.330	0.965	0.956	1.913
F (000)	268	1256	660	684
Size (mm^3)	0.28×0.14×0.02	0.40×0.12×0.11	0.30×0.26×0.20	0.11×0.07×0.02
θ range (°)	2.565 to 30.286	2.367 to 30.946	2.317 to 30.402	2.861 to 77.775
R. C.	21817	184638	62557	38401
I. R.	2677	8024	7525	5687
Goodness	1.086	1.048	1.076	1.041
R [I>2α(I)]	2483	7307	6597	5220
wR2	0.0608	0.0716	0.0871	0.1083

Table S2. Bond lengths and angles of complexes [Zn(L^1)$_2$(H$_2$O)$_2$] **(2)**, [Zn(L^1)$_2$(TMEDA)] **(3)**, [Zn(L^1)$_2$(bipy)] **(4)** and [Zn(L^1)$_2$(*o*-phen)] **(5)**

Å	**2**	**3**	**4**	**5**
Zn-O1	2.053	2.066	2.106	2.071
Zn-O2	2.053	2.066	2.058	2.071
Zn-O3	2.095	2.086	2.069	2.067
Zn-O4	2.095	2.086	2.125	2.133
Zn-O5	2.120	-	-	-
Zn-O6	2.120	-	-	-
Zn-N1	-	2.198	2.119	2.137
Zn-N2	-	2.198	2.123	2.148
°	**2**	**3**	**4**	**5**
O1-Zn-O3	87.04	85.55	85.76	86.59
O2-Zn-O3	92.96	89.67	95.85	97.25
O1-Zn-O4	92.96	89.67	170.02	168.38
O2-Zn-O4	87.04	85.55	84.62	84.45
O1-Zn-O5	90.38	-	-	-
O2-Zn-O5	89.62	-	-	-
O1-Zn-O6	89.62	-	-	-
O2-Zn-O6	90.38	-	-	-

O3-Zn-O5	88.02	-	-	-
O4-Zn-O5	91.98	-	-	-
O3-Zn-O6	91.98	-	-	-
O4-Zn-O6	88.02	-	-	-
O1-Zn-O2	180	175.53	86.16	84.83
O3-Zn-O4	180	92.25	91.38	90.33
O1-Zn-N1	-	88.56	87.55	91.06
O2-Zn-N1	-	96.59	96.01	94.37
O1-Zn-N2	-	96.59	105.06	105.88
O2-Zn-N2	-	88.56	166.40	167.17
O3-Zn-N1	-	172.50	165.96	167.02
O3-Zn-N2	-	92.38	92.69	90.55
O4-Zn-N2	-	172.50	84.61	85.34
O4-Zn-N1	-	92.38	97.20	94.38
N1-Zn-N2	-	83.67	77.15	77.82

Optical properties

ATR-FTIR Spectra

L[1]H

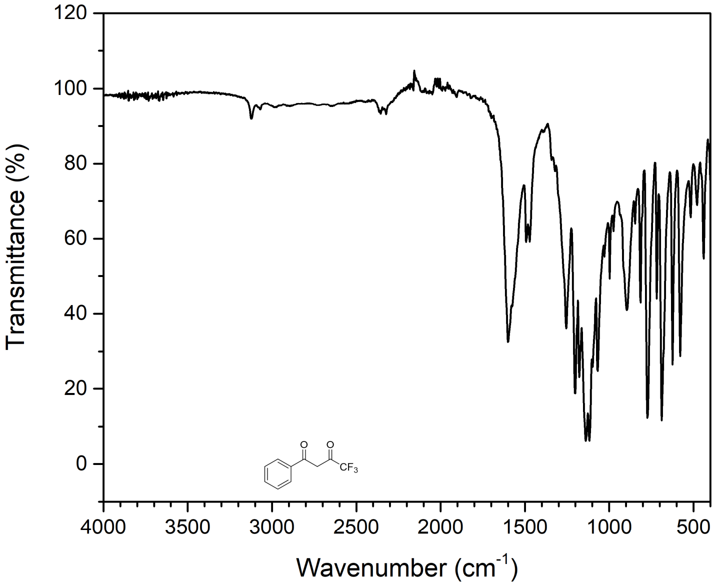

Figure S1. Infrared spectrum of ligand **L^{1}H**

Complex **2**

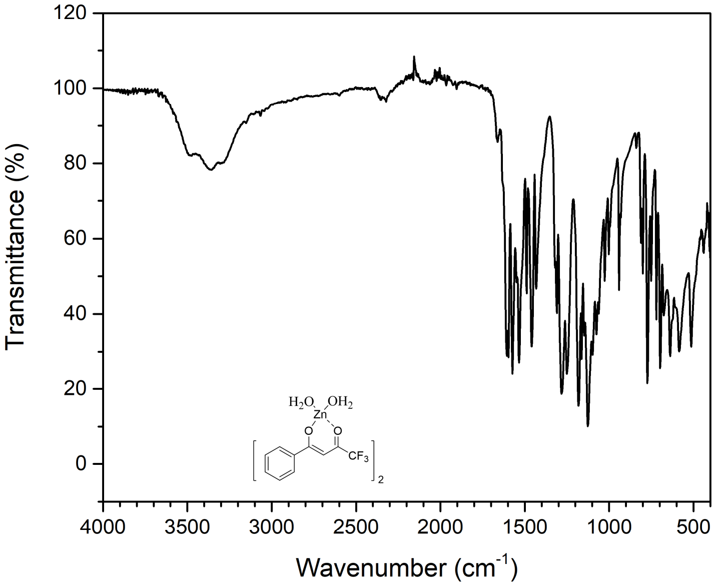

Figure S2. Infrared spectrum of complex **2**

Complex **3**

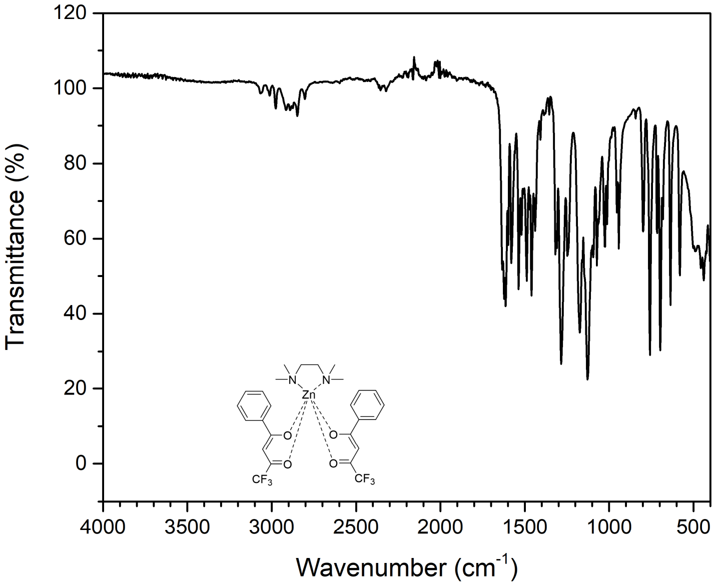

Figure S3. Infrared spectrum of complex **3**

Complex **4**

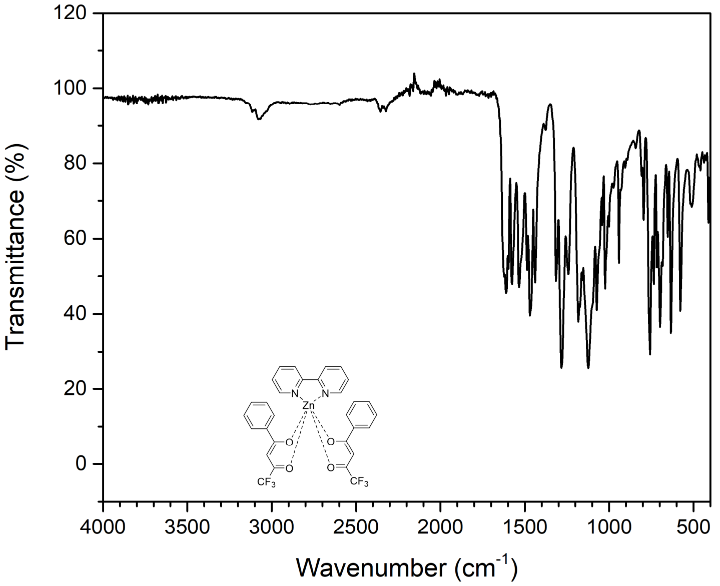

Figure S4. Infrared spectrum of complex **4**

Complex **5**

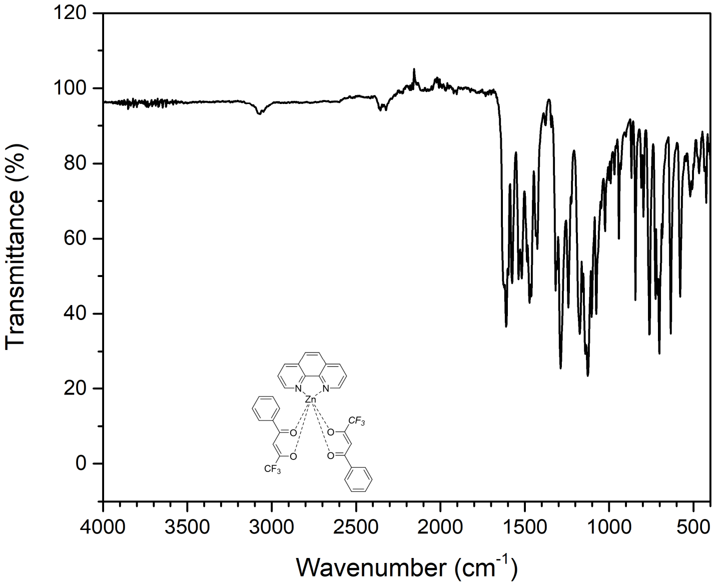

Figure S5. Infrared spectrum of complex **5**

Thermal analysis (mass loss (%) vs. temperature (°C)), under N_2 atmosphere, sample mass = 10–12 mg

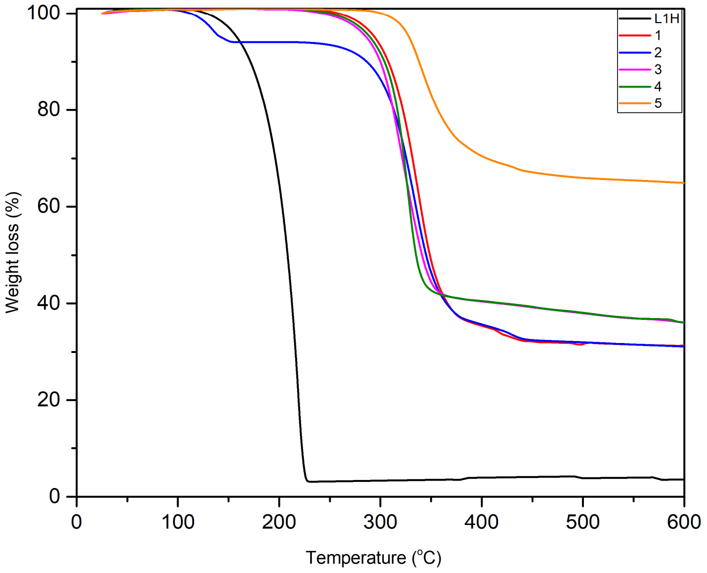

Figure S6. The thermal property (TGA) study of the ligand (**L^{1}H**) and its corresponding complexes (**1-5**)

Differential Scanning Calorimetry thermograms of complexes 2 and 5 (top to bottom: first heating, cooling, second heating) recorded from 25 to 220 °C under N_2 (30 mL min^{-1}) at 10 °C min^{-1} using a 40 µL aluminum pan

Complex **2**

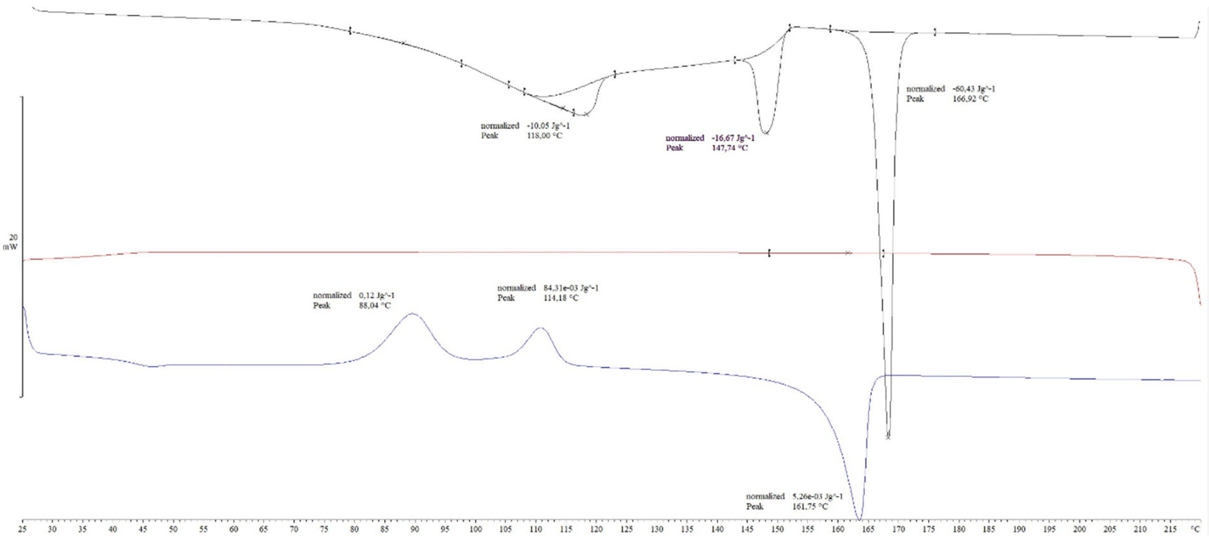

Figure S7. DSC thermogram of complex **2:** first heating (black) — dehydration at 118 °C and 147 °C, melting at 167 °C; cooling (red); second heating (blue) — crystallization at 88 °C and 114 °C, melting at 162 °C. m = 7.13 mg

Complex **5**

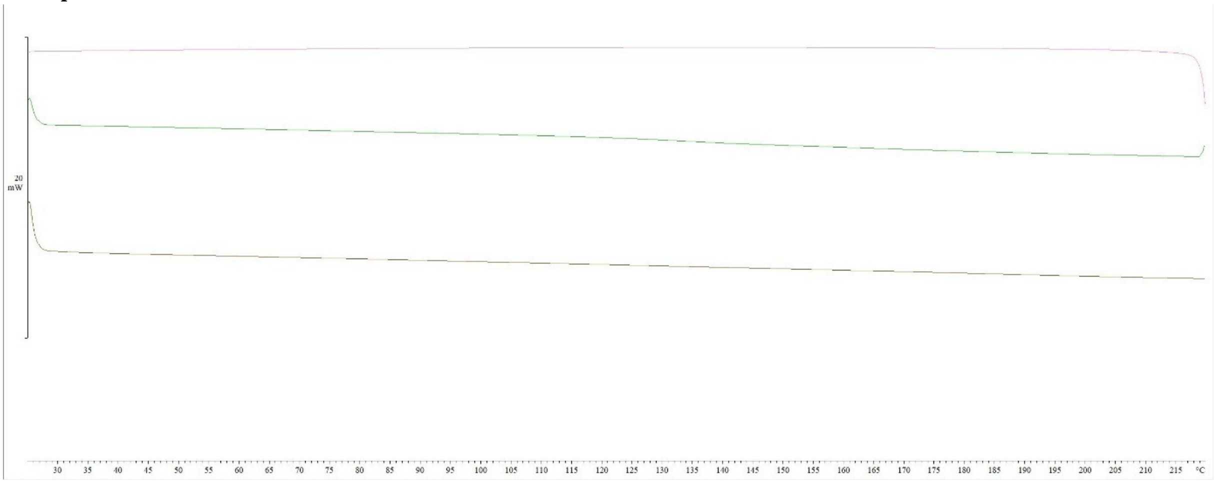

Figure S8. Differential Scanning Calorimetry (DSC) thermogram of complex **5.** The first heating (pink), cooling (green), and second heating (black) scans show no detectable thermal transitions. Sample mass: 6.4 mg

ESI Mass Spectrometry (mass range 50–1500 m/z): nebulizer pressure = 0.3 bar, source temperature = 200 °C, capillary voltage = 2500 V, dry gas flow = 4.0 L min^{-1}, end plate offset = –500 V

Complex **2**

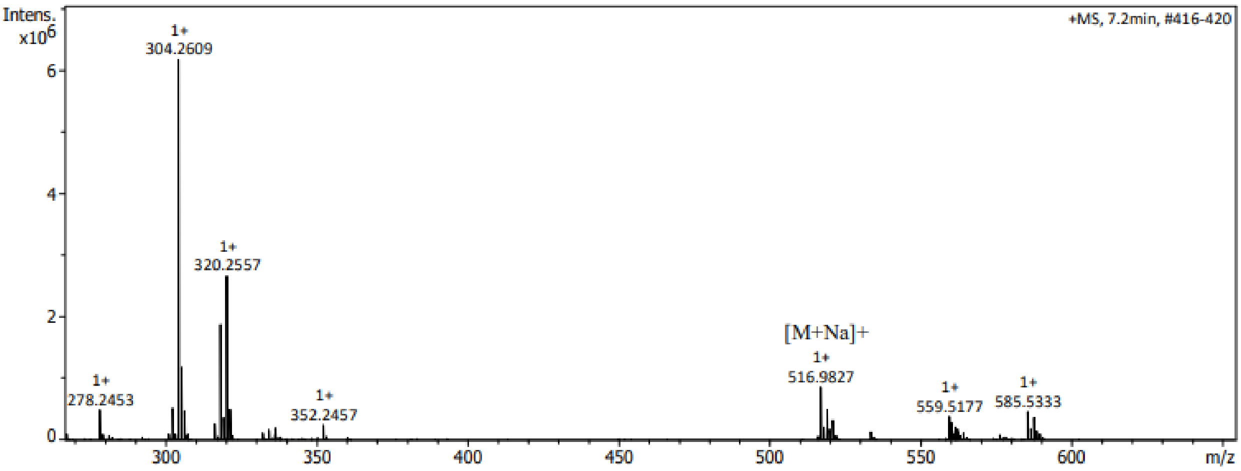

Figure S9. The mass spectrometry of complex **2**

Complex **3**

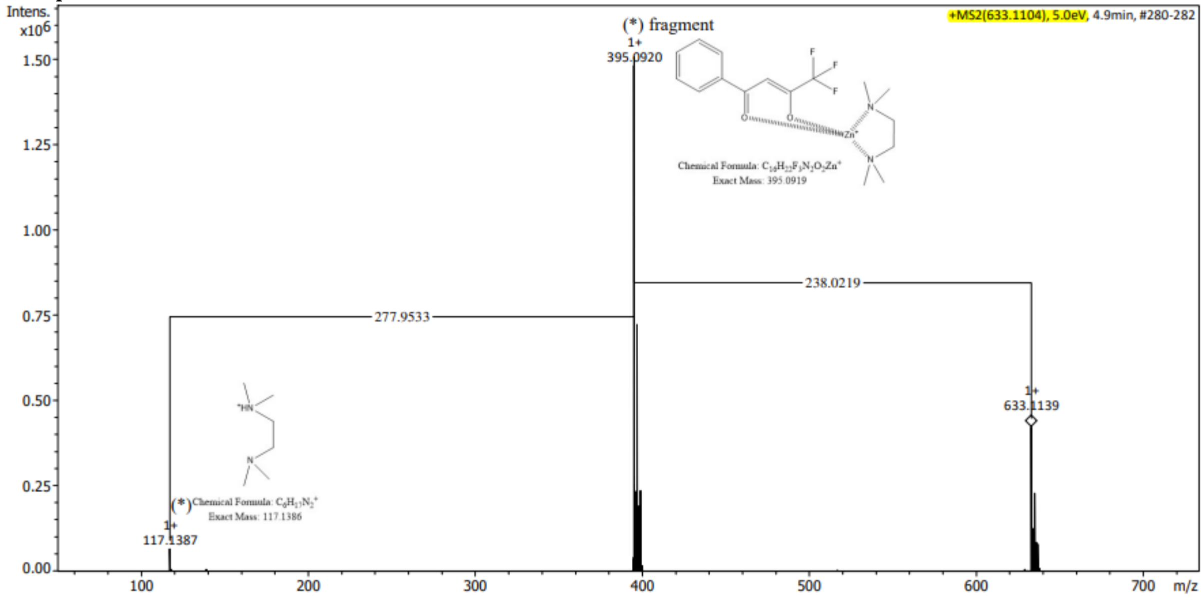

Figure S10. The mass spectrometry of complex **3**

Complex **4**

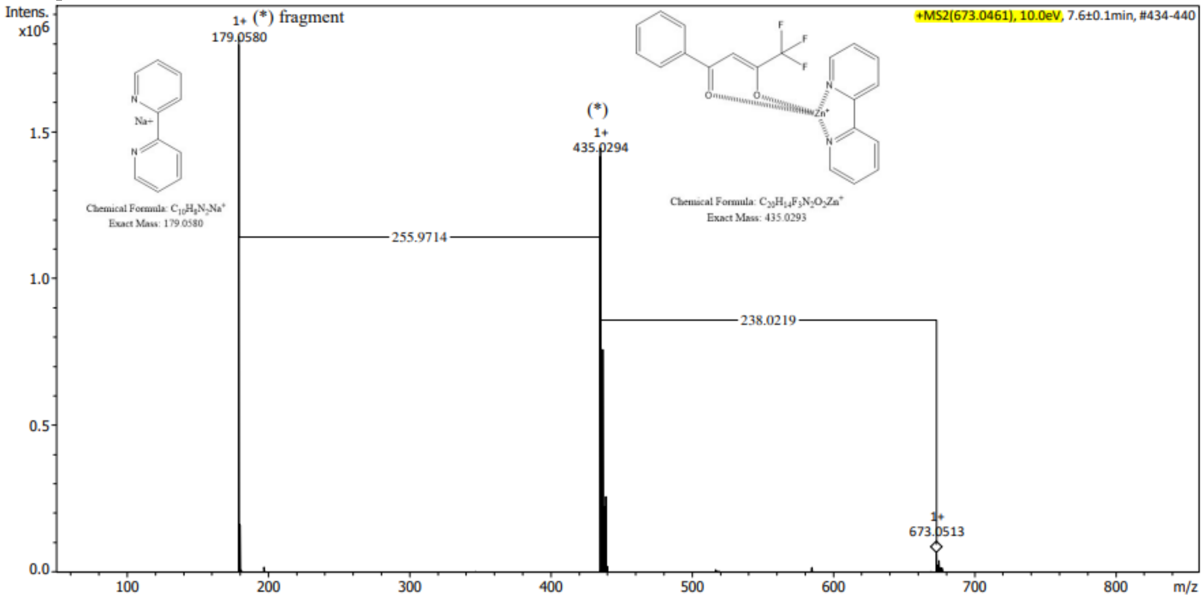

Figure S11. The mass spectrometry of complex **4**

Complex **5**

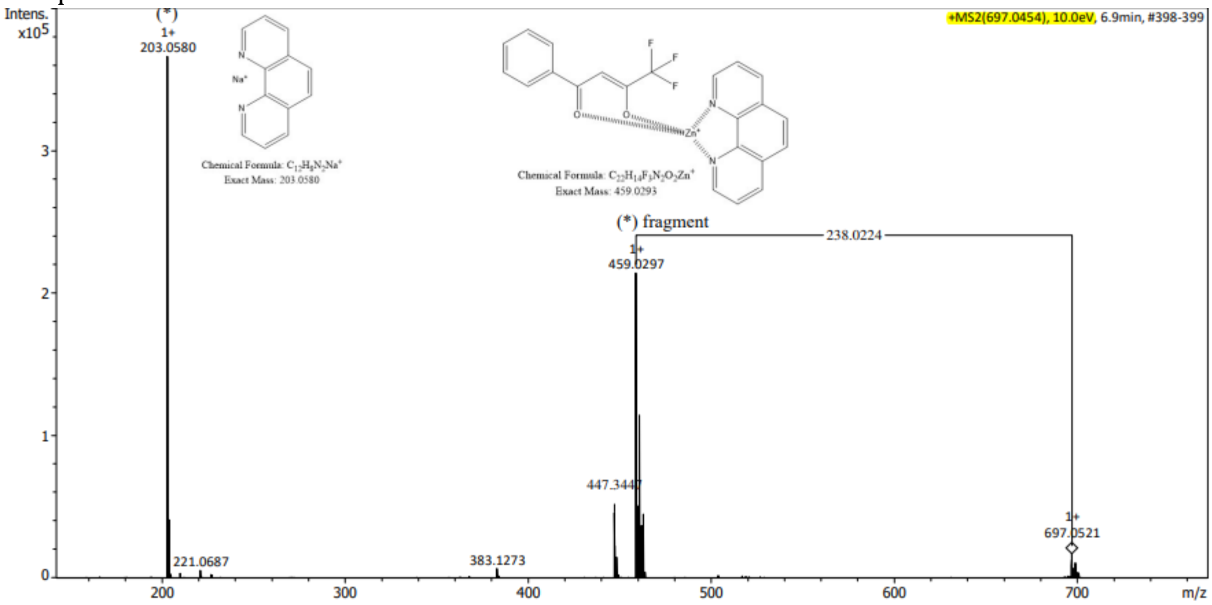

Figure S12. The mass spectrometry of complex **5**

UV–Vis (Absorbance vs. wavelength (nm)), concentration =10^{-5} M, quartz cell, path length = 1 cm.

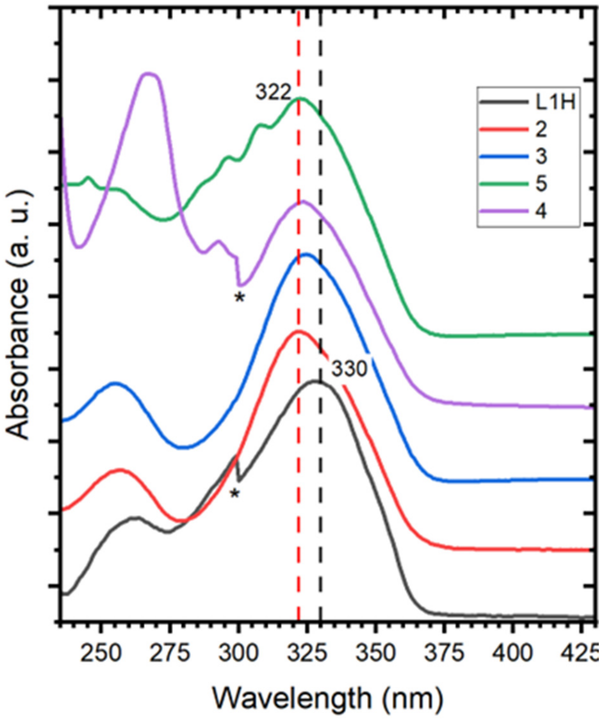

Figure S13. The UV-vis study of the ligand (**L¹H**) and its corresponding complexes (**2-5**)

Femtosecond transient absorption spectroscopy (TAS)

TAS Spectra

Complex **3**

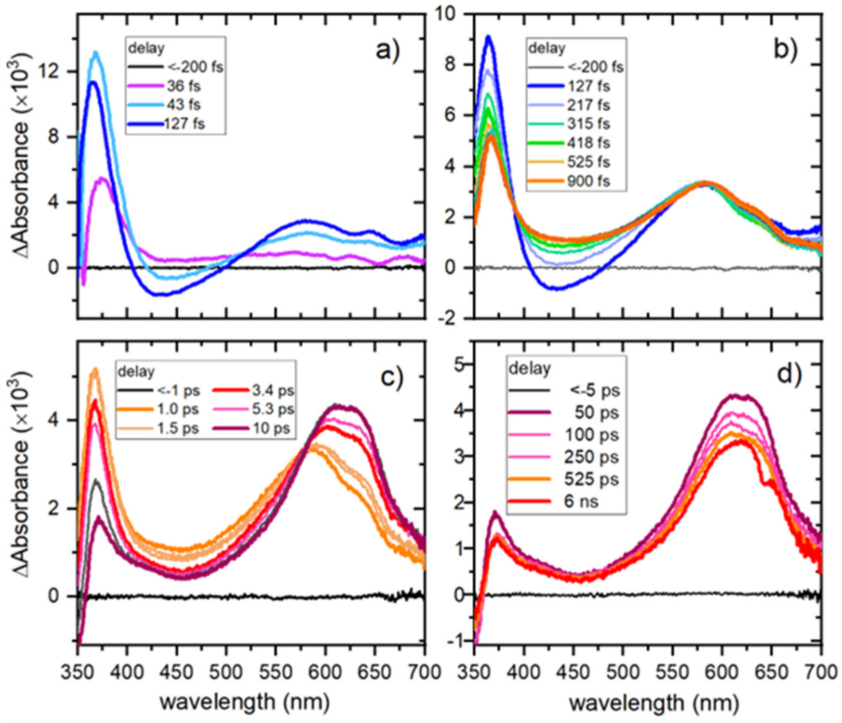

Figure S14. Transient absorption spectra of complex [Zn(L¹)₂(TMEDA)] **(3)** recorded in ethanol with a pump excitation λ_{pump} = 320 nm

Complex **4**

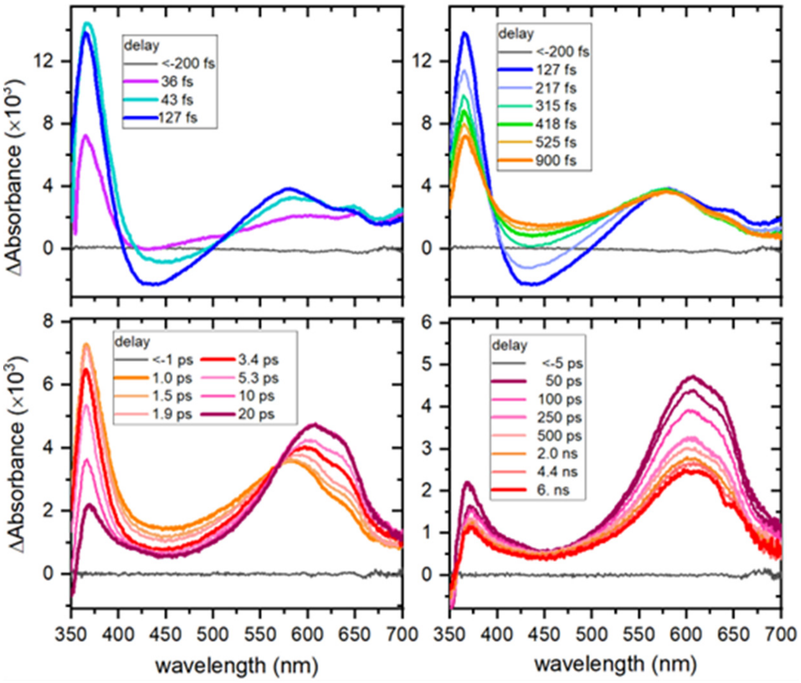

Figure S15. Transient absorption spectra of compounds [Zn(L¹)₂(bipy)] **(4)** recorded in ethanol with a pump excitation λ_{pump} = 320 nm

Complex **5**

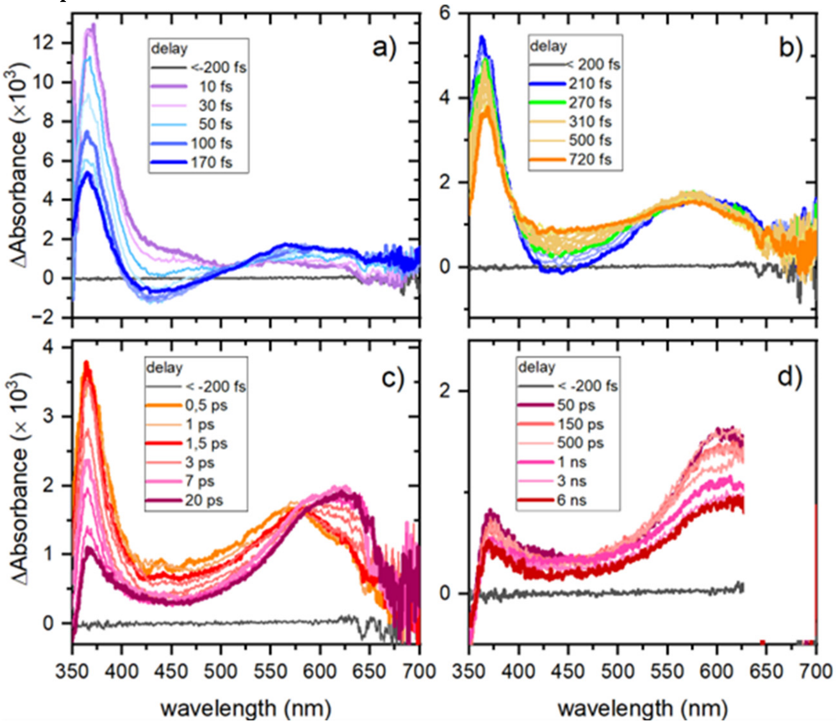

Figure S16. Transient absorption spectra of compounds [Zn(L^1)$_2$(o-phen)] **(5)** recorded in ethanol with a pump excitation λ_{pump} = 320 nm

fs/ ps Decays Associated Spectra and kinetics

The principal kinetic traces of the TAS data have been calculated by Single Value Decomposition (SVD) of the time-dependent transient spectra, and a global fit was applied to these principal kinetic components to calculate the decay associated species (DAS). All these calculations were performed using the Surface Explorer software. The results for the complexes 2-5 are displayed in the **figures S17-S20**. In addition, **figure S21** compares the DAS of the long-living species (> ns). The decay times from obtained using a 4-exponential fitting function are reported together in **table 3**.

Complex **2**

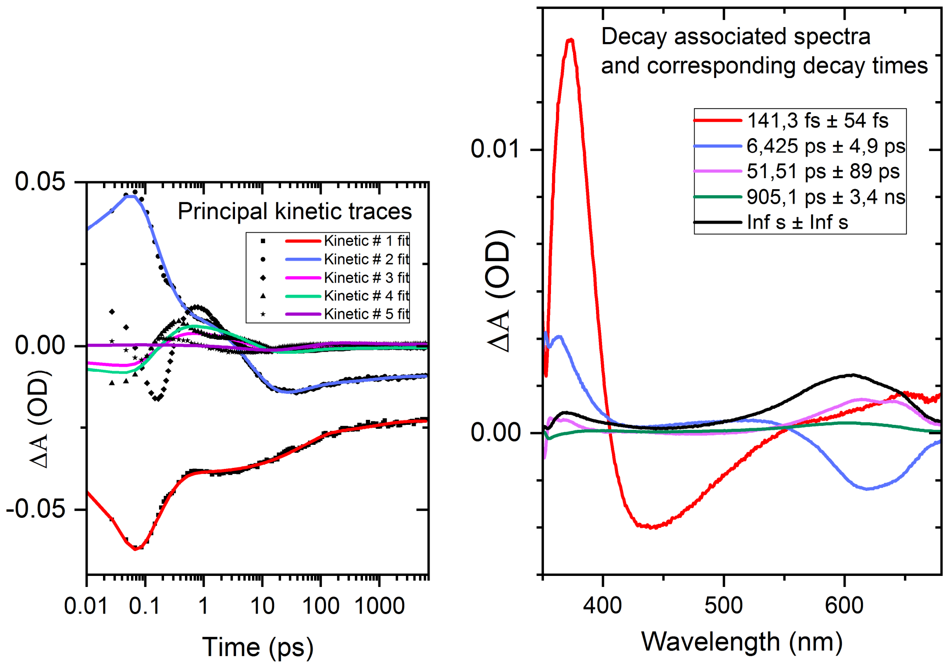

Figure S17. Principal kinetic traces (left) and decay associated spectra and their decay times (right) calculated from the TAS data of complex $[Zn(L^1)_2(H_2O)_2]$ **(2)**

Complex **3**

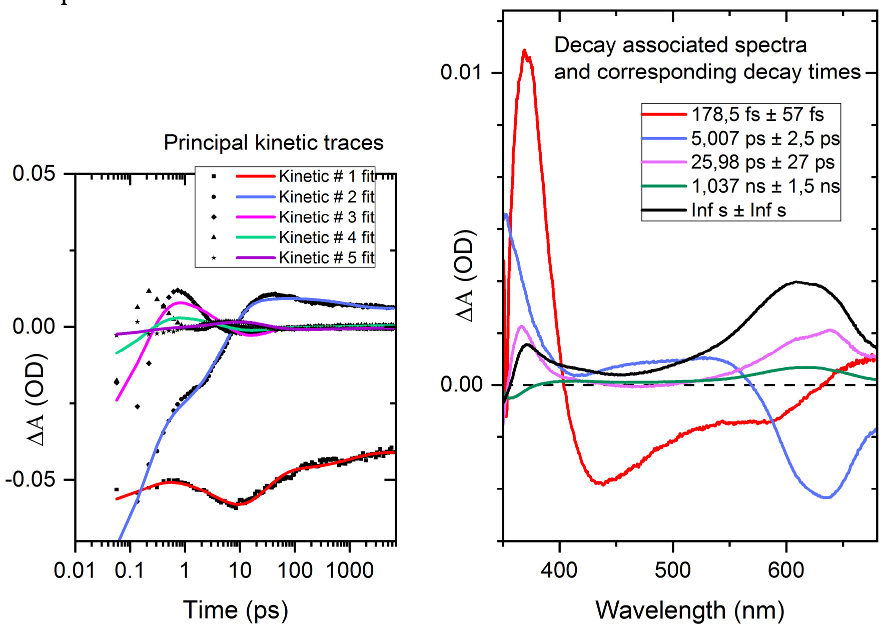

Figure S18. Principal kinetic traces (left) and decay associated spectra and their decay times (right) calculated from the TAS data of complex [Zn(L^1)$_2$(TMEDA)] **(3)**

Complex **4**

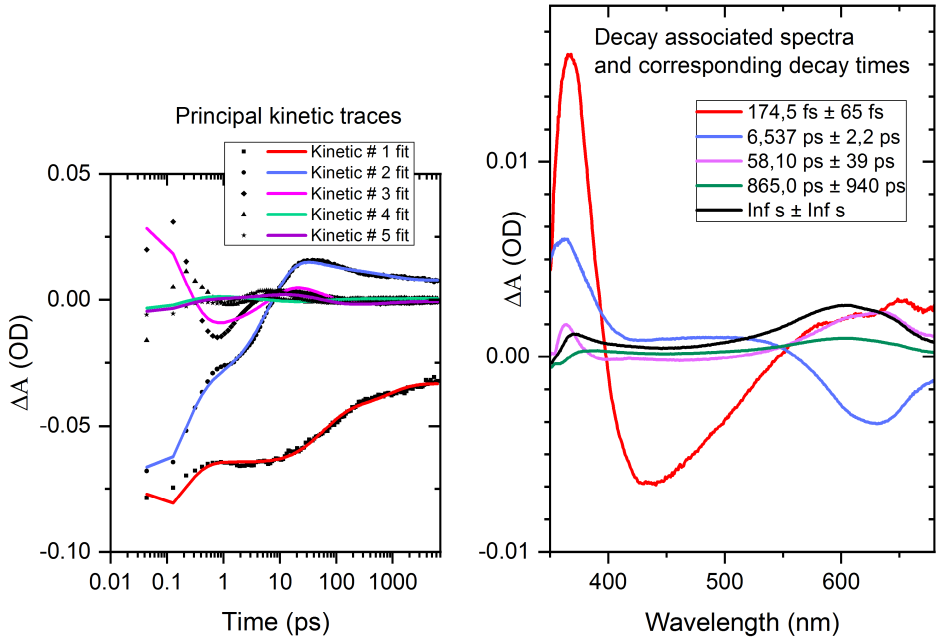

Figure S19. Principal kinetic traces (left) and decay associated spectra and their decay times (right) calculated from the TAS data of complex [Zn(L^1)$_2$(bipy)] **(4)**

Complex **5**

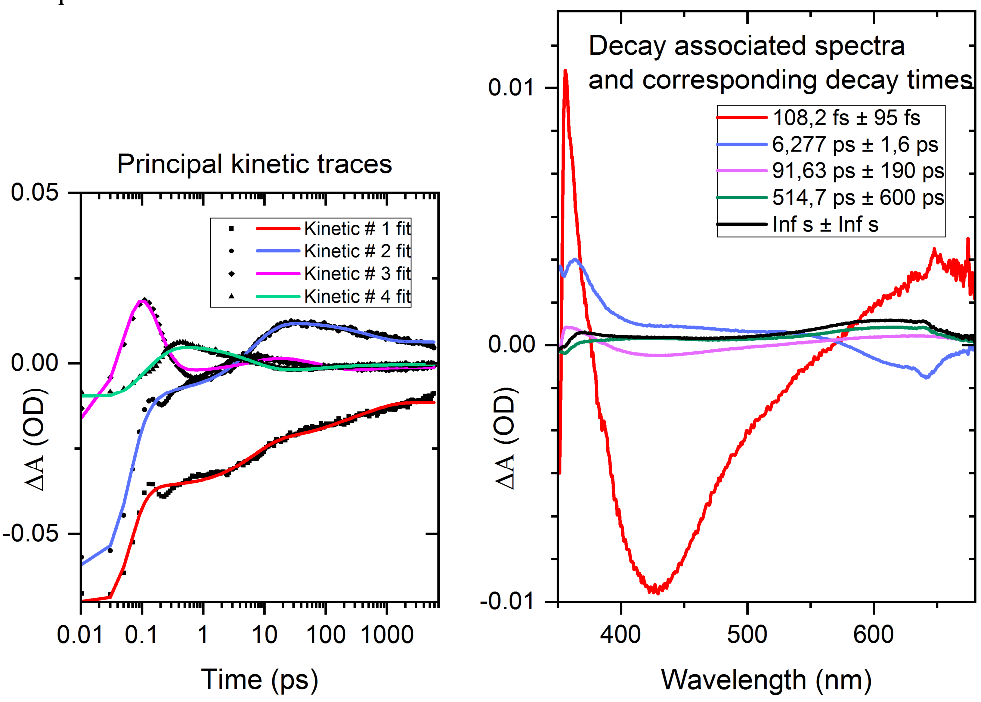

Figure S20. Principal kinetic traces (left) and decay associated spectra and their decay times (right) calculated from the TAS data of complex [Zn(L[1])$_2$(o-phen)] **(5)**

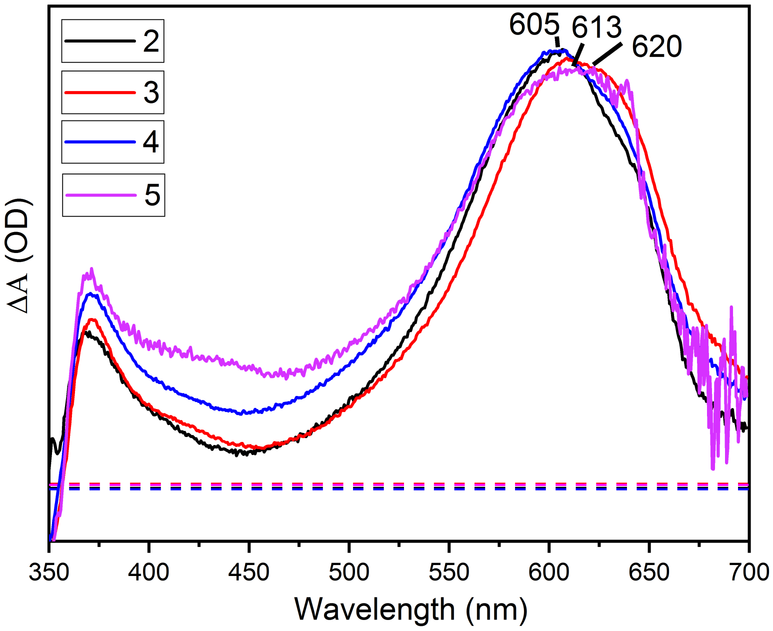

Figure S21. Normalized decay associated spectra of the long living species observed in the TAS data of the complex **2-5**

Table S3. The decay data for each complex are presented in **figure S21**

Complex	t1	t2	t3	t4
**2**	141 fs	6.4 ps	51 ps	0.9 ns
**3**	178 fs	5 ps	26 ps	1 ns
**4**	174 fs	6.5 ps	58 ps	0.86 ns
**5**	100 fs	6.2 ps	91 ps	0.5 ns

Table S4. C,H,N elemental Analysis for complexes **2-5**

Complex	E.A.	% C	% H	% N
$C_{20}H_{16}F_6O_6Zn$ **(2)**	*Calcd*	*45.14*	*3.01*	----
MW = 531.7 g.mol^{-1}	Exp.			----
$C_{26}H_{28}N_2F_6O_4Zn$ **(3)**	*Calcd*	*50.99*	*4.58*	*4.58*
MW = 611.87 g.mol^{-1}	Exp.			
$C_{30}H_{20}N_2F_6O_4Zn$ **(4)**	*Calcd*	*55.23*	*3.07*	*4.30*
MW = 651.85 g.mol^{-1}	Exp.			
$C_{32}H_{20}N_2F_6O_4Zn$ **(5)**	*Calcd*	*56.82*	*2.96*	*4.14*
MW = 675.87 g.mol^{-1}	Exp.			
